# Rok from *B. subtilis*: Bridging genome structure and transcription regulation

**DOI:** 10.1111/mmi.15250

**Published:** 2024-03-21

**Authors:** Amanda M. Erkelens, Bert van Erp, Wilfried J. J. Meijer, Remus T. Dame

**Affiliations:** ^1^ Leiden Institute of Chemistry, Leiden University Leiden the Netherlands; ^2^ Centre for Microbial Cell Biology Leiden University Leiden the Netherlands; ^3^ Centre for Interdisciplinary Genome Research Leiden University Leiden the Netherlands; ^4^ Centro de Biología Molecular Severo Ochoa (CSIC‐UAM) C. Nicolás Cabrera 1, Universidad Autónoma Madrid Spain; ^5^ Present address: Department of Human Genetics Leiden University Medical Center Leiden the Netherlands

**Keywords:** architectural proteins, bacterial chromatin, genome organization, nucleoid, transcriptional regulation

## Abstract

Bacterial genomes are folded and organized into compact yet dynamic structures, called nucleoids. Nucleoid orchestration involves many factors at multiple length scales, such as nucleoid‐associated proteins and liquid–liquid phase separation, and has to be compatible with replication and transcription. Possibly, genome organization plays an intrinsic role in transcription regulation, in addition to classical transcription factors. In this review, we provide arguments supporting this view using the Gram‐positive bacterium *Bacillus subtilis* as a model. Proteins BsSMC, HBsu and Rok all impact the structure of the *B. subtilis* chromosome. Particularly for Rok, there is compelling evidence that it combines its structural function with a role as global gene regulator. Many studies describe either function of Rok, but rarely both are addressed at the same time. Here, we review both sides of the coin and integrate them into one model. Rok forms unusually stable DNA–DNA bridges and this ability likely underlies its repressive effect on transcription by either preventing RNA polymerase from binding to DNA or trapping it inside DNA loops. Partner proteins are needed to change or relieve Rok‐mediated gene repression. Lastly, we investigate which features characterize H‐NS‐like proteins, a family that, at present, lacks a clear definition.

## Graphical abstract

In our review on the dual role of *B. subtilis* Rok as genome organizer and gene regulator, we argue that these two functions are in fact two sides of the same coin and that the coupling between them should be taken into account in further research. We also propose a list of characteristics to aid in determining whether a protein belongs to the H‐NS‐like protein family, which, at present, lacks a clear definition.

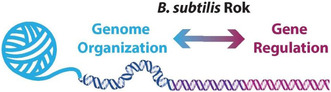

## 1 INTRODUCTION

Every organism in the tree of life faces the same problem: the effective volume of its genome far exceeds the volume of the cell or cellular compartment in which it is contained. To fit, a genome must be highly organized and compacted. Simultaneously, the DNA must remain accessible for genomic transactions such as transcription and replication. The main factors contributing to genome organization are DNA supercoiling, macromolecular crowding and binding of chromatin proteins with architectural properties (Dame, 2005; Luijsterburg et al., 2006, 2008; Stuger et al., 2002).

In eukaryotes, genome organization leads to the formation of topologically associated domains (TADs) at the kilo‐ to mega‐basepair scale (Dixon et al., 2012; Nora et al., 2012; Sexton et al., 2012). TADs are self‐interacting regions of the chromosome that are insulated from other parts of the genome (reviewed in da Costa‐Nunes & Noordermeer (2023); Szabo et al. (2019)). Structural maintenance of chromosome (SMC) proteins play a role in the formation of TADs by loop extrusion (Alipour & Marko, 2012; Fudenberg et al., 2016; Sanborn et al., 2015). Highly transcribed genes, such as tRNAs and other housekeeping genes, and insulator proteins often constitute the borders of TADs (Sexton et al., 2012).

The bacterial analogues of TADs are called chromosome interaction domains (CIDs) whose sizes range between tens and hundreds of kbps within a genome of, in the case of *B. subtilis*, 4.2 Mbps (Dame et al., 2020; Kunst et al., 1997). Both TADs and CIDs are nested structures: they consist of several smaller subdomains, the size of an operon or a few genes, that together form the larger TAD or CID (Hsieh et al., 2015; Le et al., 2013; Lioy et al., 2018; Marbouty et al., 2015; Rao et al., 2014; Wang et al., 2018).

The smallest structural unit may correspond to loops formed at individual genes or operons by architectural chromatin proteins (Hsieh et al., 2015).

In bacteria, architectural proteins are collectively referred to as nucleoid‐associated proteins (NAPs), which can be classified based on their architectural properties: DNA wrapping, bending, bridging or formation of a nucleoprotein filament (Luijsterburg et al., 2006, 2008). Besides affecting the structure of a genome, NAPs often also play roles in transcription, replication or segregation. At least 12 proteins have been classified as NAPs in the Gram‐negative model organism *Escherichia coli* (Azam & Ishihama, 1999). These include MukBEF, which is part of the structural maintenance of chromosomes (SMC) protein family (Danilova et al., 2007; Hiraga et al., 1989; Niki et al., 1991) and the DNA bending proteins HU, IHF and Fis (Pan et al., 1996; Rice et al., 1996; Swinger et al., 2003). Another *E. coli* NAP is the histone‐like nucleoid structuring protein (H‐NS), which has an overarching role in *E. coli* in global gene regulation (particularly horizontally acquired genes) and genome organization by both binding along DNA to form a nucleoprotein filament and by bridging DNA (Amit et al., 2003; Dame et al., 2000, 2006; Hommais et al., 2001). *Bacillus subtilis* is the Gram‐positive equivalent of *E. coli* as a model organism and is used to study fundamental processes like genome replication, cell division and differentiation processes like natural transformation and sporulation. Also, *B. subtilis* is an important industrial organism because of its ability to secrete proteins and other chemical compounds (Errington & van der Aart, 2020). Many *E. coli* NAPs have a homolog in *B. subtilis*: BsSMC and HBsu are homologs of the *E. coli* MukBEF and HU proteins, respectively (Micka et al., 1991; Moriya et al., 1998; White et al., 1989). Rok (repressor of *comK*) has been proposed to be the H‐NS analogue in *B. subtilis* based on its DNA binding preferences and its role in silencing horizontally acquired genes (Hoa et al., 2002; Smits & Grossman, 2010). Additionally, Rok regulates other genes encoding cell surface and extracellular proteins and those encoding antimicrobial compounds (Albano et al., 2005; Denham et al., 2019). More recently, it was found that Rok also plays a role in genome organization by establishing long‐range chromosomal loops, most likely by DNA bridging (Dugar et al., 2022; Erkelens et al., 2022; Marbouty et al., 2015). A dual function in genome organization and transcription regulation has also been observed for other NAPs (Amemiya et al., 2021; Qin et al., 2019; Singh et al., 2016; Winardhi et al., 2015), but the interplay between the structural and regulatory roles of these proteins has received little attention. Here, we address this topic by connecting the disparate studies on these two processes with a focus on Rok. We propose that the DNA bridging ability of Rok is not only crucial for its role in genome architecture but also forms the basis for its transcriptional role, aided by partner proteins that finetune the repressive impact.

## 2 STRUCTURE AND ORGANIZATION OF THE *B. subtilis* GENOME

The chromosome of *B. subtilis* is organized at different length scales from a global genome level of Mbps down to operon and gene level from Kbps to hundreds of bps. At the chromosome‐wide scale, chromosome conformation capture techniques, such as 3C and Hi‐C, provide information on the frequency of interactions between loci in three‐dimensional space, that can be visualized as two‐dimensional heat maps (Crémazy et al., 2018; Dekker et al., 2002; Lieberman‐Aiden et al., 2009; van Berkum et al., 2010). Chromosomal positions that are sequentially close to each other have a higher probability of interacting. Hence, these maps exhibit a diagonal of high intensity (Dekker et al., 2002).

For *B. subtilis*, but not for *E. coli*, a second diagonal is visible in Hi‐C maps perpendicular to the main diagonal (Lioy et al., 2018; Marbouty et al., 2015). This second diagonal is interpreted as reflective of the left‐ and right‐chromosomal arms being zipped together in *B. subtilis*. High DNA density areas are also observed around the origin of replication (*ori*) and, to a lesser extent, at the terminus region (*ter*) (Marbouty et al., 2015; Tišma et al., 2023). At a scale of tens to hundreds of kbps, CIDs are formed, which are visible as squares along the primary diagonal in Hi‐C maps (Marbouty et al., 2015). The chromosome of *B. subtilis* during exponential phase is organized into 20 CIDs with lengths ranging between 50 and 300 kb. Several *B. subtilis* NAPs, such as the BsSMC and Rok proteins have been implied in CID formation (Marbouty et al., 2015). At the gene and operon level, within the nested structure of CIDs, NAPs form local structures such as loops and filaments by bending, bridging or multimerizing along the DNA (Dame et al., 2020).

### 2.1 Nucleoid‐associated proteins (NAPs)

#### 2.1.1 SMC

**FIGURE 1:**
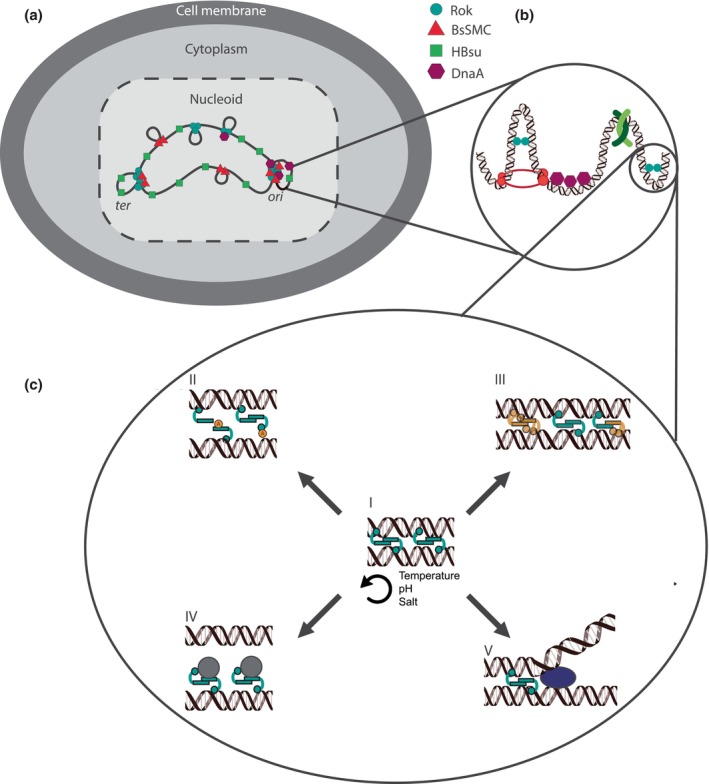
The *B. subtilis* chromosome is organized at multiple scales where Rok forms DNA–DNA bridges that can be modulated by partner proteins. (a) The genome is compacted into the nucleoid (light gray within the dotted line) and separated from the cytosol (gray). Rok (cyan circles) forms several (super)clusters around the chromosome. BsSMC (red triangles) is responsible for aligning the two chromosomal arms into a crescent shape and forms loops in the DNA. HBsu (green squares) binds along the whole chromosome. DnaA (purple hexagon) is not an architectural chromatin protein itself, but (potentially) interacts with Rok and HBsu. (b) These four proteins (among others) are all present around the *ori* region. Their local structuring properties are highlighted with Rok bridging two DNA duplexes, BsSMC forming DNA loops and HBsu bending the DNA. DnaA is abundant as a replication initiator in this region. (c) DNA binding activities and features of Rok. (I) Rok (in cyan) forms DNA–DNA bridges as dimer. This bridge is unusually stable and only mildly responsive to temperature, pH and salt concentration. (II) Rok acetylation might influence DNA binding (K142‐Ac) or dimer formation (K51‐Ac), hampering bridge formation. (III) Rok and sRok (in orange) can form homo‐ and heterodimers in vitro. While they both bridge DNA, sRok is influenced by salt and can potentially influence the stability of a DNA‐Rok:sRok‐DNA bridge. (IV) Other protein partners may bind to Rok and hinder bridge formation due to steric hindrance. (V) Without direct interaction with Rok a protein partner may have an antagonistic effect potentially via DNA topology or by disrupting a DNA–DNA bridge.

The only architectural chromatin protein family conserved throughout the tree of life is the SMC family of proteins (Cobbe & Heck, 2004). These proteins structure the chromosome by bridging two DNA segments followed by loop extrusion at the expense of ATP hydrolysis (Figure 1a,b) (Brandão et al., 2021; Gogou et al., 2021; Haering et al., 2004; Lammens et al., 2004). SMC proteins have a modular composition in which a hinge dimerization domain and an ATPase head domain are separated by an anti‐parallel coiled‐coil arm (Anderson et al., 2002; Melby et al., 1998). The *B. subtilis* SMC protein (BsSMC) is required for the segregation of newly replicated chromosomes by condensing, and thereby restraining, both DNA segments (Gruber et al., 2014; Moriya et al., 1998; Wang et al., 2014). Cells lacking BsSMC or its partner proteins ‘segregation and condensation proteins A and B’ (ScpA/B) only survive under slow‐growth conditions (Moriya et al., 1998; Soppa et al., 2002). The second diagonal in Hi‐C maps (see above) is not observed in the absence of BsSMC, strongly indicating that BsSMC is responsible for bridging and aligning the two chromosome arms (Figure 1a) (Marbouty et al., 2015). Indeed, it has recently been shown that opening up the BsSMC arms by disrupting ScpA changes the shape of the *B. subtilis* chromosome from crescent to toroidal in vivo (Tišma et al., 2023). BsSMC is particularly enriched around the highly structured *oriC* region due to its interaction with the *parABS* segregation system (Bock et al., 2022; Guilhas et al., 2020; Sullivan et al., 2009). It is not known whether BsSMC activity on DNA involves loop extrusion. Together, these proteins are key players in chromosome organization, DNA replication and DNA segregation during cell division (Roberts, 2023; Roberts et al., 2022). Reviewing these proteins in detail is beyond the scope of this review, and the reader is referred to Jalal & Le (2020) and Kim et al. (2023).

#### 2.1.2 HBsu

The non‐specific DNA binding protein HU from *B. subtilis* (HBsu) bends DNA in vitro (Figure 1b) (Köhler & Marahiel, 1997; White et al., 1989), explaining why it compacts DNA in vivo (Köhler & Marahiel, 1997). Contrary to its *E. coli* equivalent HU, HBsu is essential for cell growth and survival (Micka et al., 1991; Micka & Marahiel, 1992). The essentiality of HBsu can be explained, at least in part, by its function in replication initiation. While HBsu is required to start DNA replication from wild type *oriC*, it is not needed when *oriC* is replaced with the origin of replication *oriN* of a *B. subtilis* plasmid (Karaboja & Wang, 2022). As replication from *oriN*, unlike that from *oriC*, does not require DnaA, HBsu may act via DnaA. In *E. coli*, indeed association of HU with DnaA has been demonstrated (Chodavarapu et al., 2008), but information on physical interaction is lacking for *B. subtilis* HBsu. However, as *E. coli* HU is not essential, interaction with DnaA is probably not the only reason for the HBsu to be essential. The difference in essentiality might be explained by the fact that *E. coli* has two homologs of HU (HUα and HUβ), whereas *B. subtilis* only has HBsu (Grove, 2011).

Genome compaction by HBsu is regulated by post‐translational modifications (PTMs) on HBsu. Strains expressing mutants that mimic HBsu acetylation exhibit larger nucleoids compared to wildtype strains, suggesting that acetylation of K80 inhibits DNA binding and compaction of the genome (Carabetta et al., 2019). Several other lysines of HBsu can also be acetylated in different stages of the cell cycle (Carabetta et al., 2016). In addition, HBsu residues T4 and R61, which are close to the DNA binding site in the *E. coli* and *Anabaena* sp. HU‐DNA complexes, are susceptible to phosphorylation (Elsholz et al., 2012; Hammel et al., 2016; Macek et al., 2007; Swinger et al., 2003). However, the effects of these modifications on DNA binding and compaction are currently unknown.

#### 2.1.3 Rok

Although HBsu is distributed uniformly across the *B. subtilis* genome, Rok is only associated with parts of the genome (Figure 1a). In vivo microscopy studies, supported by ChIP‐seq data and Hi‐C experiments, show that Rok forms clusters at specific chromosomal loci (Dugar et al., 2022; Marbouty et al., 2015; Smits & Grossman, 2010). Rok co‐localizes with 30% of the CID boundaries in Hi‐C maps of *B. subtilis*, supporting the view that Rok clusters have a function in genome organization (Marbouty et al., 2015). During the stationary phase, these Rok clusters interact to form superclusters, two of which are located near the *ori* and *ter* regions (Figure 1a) (Dugar et al., 2022). While cluster formation depends on Rok, other proteins, like BsSMC, are also required for the formation of Rok clusters (Dugar et al., 2022). It is currently unknown whether BsSMC and Rok physically interact. At a subset of Rok sites in ChIP‐seq experiments, Rok co‐localizes with DnaA and DnaA binding in those locations is dependent on Rok (Seid et al., 2017). But the functional implications of this are still unknown and DnaA has no effect on the formation of Rok (super)clusters (Dugar et al., 2022). Rok and DnaA are also both present around the *ori* region (Figure 1a). Here, the two proteins likely act independently because DnaA binds to the *ori* region also in the absence of Rok (Seid et al., 2017).

Rok forms DNA–DNA bridges in vitro (Figure 1b) (Erkelens et al., 2022). Combined with the local binding of Rok observed at promotor sites of several genes (Forrest et al., 2022), this suggests that Rok regulates gene expression via DNA bridging. One of the operons regulated by Rok is the *yybN* operon, whose position coincides with one of the established Rok clusters on the genome (Dugar et al., 2022). This coincidence points towards a dual role for Rok as an architectural chromatin protein and gene regulator. An open question is whether Rok multimerization is required for either of these functions. Rok dimers have been proposed as the minimal unit required for bridging DNA, but oligomeric structures have also been observed in solution (Duan et al., 2018; Erkelens et al., 2022). The first 45 amino acids of Rok are necessary for cluster formation (Dugar et al., 2022), but whether this region is responsible only for multimerization or also for dimerization is still unclear.

### 2.2 Liquid–liquid phase separation

A phenomenon that has recently gained attention in the field is liquid–liquid phase separation (LLPS). LLPS is a form of phase transition where molecules in a solution form a dense liquid droplet within a more dilute solvent. A recent review describes general aspects of LLPS (Azaldegui et al., 2021). LLPS has also emerged as an important factor in genome organization across the tree of life (reviewed in Feric and Misteli (2021)). Proteins that drive LLPS are typically modular; they contain (self)interaction and nucleic acid binding domains and often have intrinsically disordered regions (Boija et al., 2018; Dignon et al., 2020; Sanulli et al., 2019; Wang et al., 2019). For example, eukaryotic histone tails can drive LLPS and PTMs, occurring in these tails, are able to influence this behavior (Wang et al., 2019). Originally, LLPS was found to contribute to compartmentalization in eukaryotic cells (Boeynaems et al., 2018; Shin & Brangwynne, 2017); recent studies suggest LLPS is also at play in bacteria. Some examples include ribonucleoprotein granules (RNP bodies) in *Caulobacter crescentus* that form during mRNA degradation (Al‐Husini et al., 2018) and polyphosphate granules in *Pseudomonas aeruginosa* formed during stress and starvation (Racki et al., 2017).

Contrary to eukaryotic cells, the bacterial chromosome is not confined in a nucleus that is surrounded by a membrane. Yet, bacterial genomes are compact and occupy distinct regions within a cell, and it has been suggested that LLPS plays a role in the formation of the nucleoid (Azaldegui et al., 2021; Cohan & Pappu, 2020; Janissen et al., 2018; Joyeux, 2018; Ladouceur et al., 2020; Odijk, 1998).

So far, little is known about involvement of bacterial NAPs in LLPS. Several NAPs have characteristics that could potentially drive formation of condensates via LLPS, such as DNA binding and multimerization domains, but so far very few studies have been conducted to analyze this. Although not yet explicitly demonstrated in vivo, the *E. coli* Dps protein (DNA binding protein from starved cells) is a promising candidate. This protein becomes highly expressed in stationary phase cells and, upon binding to DNA, compacts the genome and protects it from damage (Ali Azam et al., 1999; Antelmann et al., 1997; Kim, 2004). Indeed, Dps and HU form condensates when mixed with DNA in vitro (Gupta et al., 2023). Because of its ability to form clusters at specific chromosomal regions and its modular composition (Duan et al., 2018; Dugar et al., 2022), Rok might also drive LLPS. To test this in vitro, pre‐formed Rok DNA–DNA bridges were challenged by addition of 1,6 hexanediol – a compound often used to dissolve LLPS condensates – but DNA bridging was unaffected (Erkelens et al., 2022). Although the obtained result suggests that Rok is not an LLPS protein, it may play such a role when combined with other protein(s). Many details are still lacking on the effects of LLPS on genome compaction and cellular processes like transcription, which is an interesting area for further research.

## 3 Rok HAS A DUAL FUNCTION AS A GENOME ORGANIZER AND GENE REGULATOR

The process of transcription is, to a large extent, conserved throughout the tree of life. Bacteria, archaea and eukaryotes all express RNA polymerase (RNAP) to transcribe DNA into RNA. A transcription event in bacteria is initiated when an RNA polymerase holoenzyme complex is recruited to a promoter by the binding of its σ factor to the promotor ‐10 element. The *B. subtilis* genome encodes many σ factors that are expressed as a function of environmental conditions. This provides specificity of RNAP binding to defined sets of promotors (reviewed in Feklístov et al. (2014) and Rodriguez Ayala et al. (2020)). For example, σ^E^, σ^F^, σ^G^ and σ^H^ are active during various stages of sporulation to switch on genes required to progress sequentially through the different stages of spore development (reviewed in Galperin et al. (2022)). The housekeeping σ^A^ factor of *B. subtilis* has greater specificity for canonical promotors than the related *E. coli* σ^70^ factor, thereby limiting spurious transcription from intragenic transcription start sites (Forrest et al., 2022). The *E. coli* and *B. subtilis* analogs H‐NS and Rok have evolved their genomic binding patterns to adapt to the different promiscuity levels of their σ factor and RNAP. While *E. coli* H‐NS forms filaments that extend into coding regions to prevent spurious transcription from intragenic promotors, Rok binds more locally to regions containing the canonical promotor sequences (Forrest et al., 2022).

Rok was originally identified as a repressor *of comK* (regulator of competence and DNA uptake) and thus, Rok is involved in the competence pathway of *B. subtilis* (Hoa et al., 2002). Other genes that are regulated by Rok, usually repressed, encode for secreted or membrane‐localized proteins (Albano et al., 2005). Examples are the secreted antimicrobial peptide Sublancin 168 and the stress‐responsive membrane protease HtpX (Denham et al., 2019; Marciniak et al., 2012). Besides being subject to direct regulation, several genes, such as *yuaB* that plays a role in biofilm formation, are indirectly regulated by Rok (Kovács & Kuipers, 2011). Due to its preference for AT‐rich DNA, Rok binds and regulates the expression of several prophages in the *B. subtilis* genome as well as other horizontally acquired genes. This classifies Rok as a xenogeneic silencer, which recognizes and represses expression from newly acquired genes. As stated above, the *yybN* operon of unknown function underlies one of the eight identified Rok clusters on the *B. subtilis* genome [cluster 8, retaining the nomenclature used in (Dugar et al., 2022)]. In earlier studies, *yybN* was proposed to be acquired via horizontal gene transfer and was found to be a direct target of Rok (Albano et al., 2005; Nicolas, 2002; Smits & Grossman, 2010). Two other clusters also contain genes that were reported previously to be regulated by Rok (*sunT* and *sunA* [cluster 3], and *sdpA/B/C* [cluster 5]). (Albano et al., 2005; Denham et al., 2019; Dugar et al., 2022). The genes in other Rok (super)clusters have not been reported to be Rok‐regulated so far. Also, many other Rok‐regulated genes are not covered in the Rok (super)clusters (Albano et al., 2005; Erkelens et al., 2022). Rok can bridge two DNA strands in vitro, and in vivo studies have shown that Rok binds locally at promotor regions often resulting in repression of those genes (Erkelens et al., 2022; Forrest et al., 2022). Therefore, it is likely that Rok interferes with transcription initiation by promotor occlusion. Indeed, the binding of Rok at the *comK* promotor region reduces the amount of RNAP that binds to the promotor (Smits et al., 2007). Rok may also repress gene expression in a completely different way. The DNA bridging ability of Rok could result in DNA loop formation which can trap RNAP at the promotor region and prevent transcription initiation (Dame et al., 2002; Forrest et al., 2022; Qin et al., 2019). Such loops that may form at the tens to a few hundred bps are, at present, smaller than the resolution of available *B. subtilis* Hi‐C maps (Dugar et al., 2022; Marbouty et al., 2015), but Rok binding at promotor regions does show up in ChIP‐seq experiments (Seid et al., 2017; Smits & Grossman, 2010). These different sensitivities may explain why Rok clusters do not fully cover all Rok‐regulated genes. Moreover, some genes may be subject to indirect regulation by Rok, due to repression of expression of other regulatory proteins.

An additional role for Rok, next to regulating *comK*, in natural transformation and horizontal gene transfer has been proposed (Serrano et al., 2021). While Rok is unable to bridge (incoming) ssDNA with dsDNA (Erkelens et al., 2022), a bridge between genomic DNA and small dsDNA structures formed due to internal hybridization of ssDNA might still be possible. As incoming ssDNA will be covered by the single‐stranded DNA‐binding proteins SsbA and SsbB (Yadav et al., 2012), leaving little place for Rok to bind and keeping the DNA single‐stranded, a direct role in this process is not likely. However, as several binding partners of Rok have been proposed (see below), we cannot rule out any functional or physical interaction between Rok and SsbA or SsbB.

## 4 THE FUNCTION OF Rok IS MODULATED BY PARTNER PROTEINS

Bacteria need to adapt to changes in living conditions and this is reflected by changes in genome organization and gene expression. Therefore, NAPs play a significant role in sensing and reacting to changing conditions. *E. coli* H‐NS switches between its DNA bridging state and nucleofilament formation in vitro in response to physico‐chemical cues (Boudreau et al., 2018; Liu et al., 2010; Qin et al., 2020; van der Valk et al., 2017). These different DNA binding states have their effect on gene regulation (Atlung et al., 1997; Göransson et al., 1990; Nagarajavel et al., 2007; Ono et al., 2005; Rashid et al., 2023; Ueguchi & Mizuno, 1993; White‐Ziegler & Davis, 2009).

The DNA bridging behavior of H‐NS is also modulated by other mechanisms like PTMs and (protein) binding partners (reviewed in (Qin et al., 2019)). Recently, also the second messenger cyclic di‐GMP was shown to affect the DNA binding (and, by extrapolation, DNA bridging) by H‐NS (Li et al., 2023). While it is very likely that Rok represses some genes by DNA bridging (see above), it is unclear how Rok‐mediated gene repression is relieved. The DNA bridging behavior of Rok is only mildly responsive to physico‐chemical perturbations (Erkelens et al., 2022). It is thus unlikely that these changing conditions have a physiological function in terms of affecting the behavior of Rok (Figure 1c). Moreover, the number of Rok molecules in *B. subtilis* grown in competence medium is rather constant throughout the cell cycle (Hoa et al., 2002). Therefore, a mechanism using targeted degradation of non‐DNA‐bound protein by, for instance, the Lon protease, as observed for H‐NS and its paralogue StpA is unlikely (Choi et al., 2022; Johansson et al., 2001; Johansson & Uhlin, 1999).

Two proteome studies in *Bacillus* sp. show that Rok can be acetylated on K51 and K142 (Liu et al., 2016; Reverdy et al., 2018). Because these residues are present in the multimerization and DNA binding domains respectively, acetylation may affect the DNA bridging by Rok (Figure 1c) (Qin et al., 2019). Functional studies investigating the possible role of acetylation of these residues are currently lacking. For H‐NS, many more PTMs have been identified on key residues for DNA binding and interaction with itself or other proteins (Dilweg & Dame, 2018). PTMs are, therefore, an important feature of global gene regulation and genome organization by NAPs in bacteria.

Although Rok itself is not a direct sensor of environmental changes (see above), a recurring theme is functional modulation via protein partners. For instance, the naturally occurring internally truncated derivative sRok interacts with Rok (Figure 1c) (Erkelens et al., 2022). sRok was first identified on the pLS20 plasmid, but is also found in various *Bacillus sp.* (Singh et al., 2012). sRok can replace Rok in the competence pathway. Taking into account that Rok and sRok exhibit 48% sequence similarity, sRok can, surprisingly, both bridge DNA and form a nucleofilament, in contrast to Rok (Erkelens et al., 2022). The DNA bridging behavior of sRok is osmoresponsive and this extends to the Rok:sRok complex. The regulon of the heteromeric complex partly overlaps with that of Rok and sRok alone, but also contains a unique set of genes not found for the two single proteins. The exact details of the interaction between Rok and sRok are still unclear, but sRok has the potential to couple Rok to changes in physico‐chemical conditions.

As described above, DnaA and Rok co‐localize on certain regions of the genome (Seid et al., 2017). DnaA binds directly to DnaA boxes at the *ori* region and near genes that are directly regulated by DnaA (Ishikawa et al., 2007). Association of DnaA with regions that do not contain DnaA boxes is dependent on the presence of Rok at those locations (Smith & Grossman, 2015). This co‐localization is not dependent on the DNA binding domain of DnaA, which indicates that DNA‐bound Rok interacts with DnaA via other parts of the protein (Seid et al., 2017). However, a direct, physical interaction between Rok and DnaA has not yet been shown. As DnaA functions as a replication initiator and is an ATPase (Kaguni, 2006; Ogura et al., 2001; Sekimizu et al., 1987), this interplay could connect Rok to both the replication and energy status of the cell. One of the operons regulated by both Rok and DnaA is *yybN* (Seid et al., 2017). This could again be indicative of structural regulation, as *yybN* is located in one of the Rok clusters (see above). It remains to be investigated if DnaA can affect Rok‐DNA bridges.

Lastly, ComK and Rok have been found to co‐localize at the *comK* promotor (Smits et al., 2007). While Rok represses the expression of *comK* together with ArbB and CodY (Hamoen et al., 2003; Hoa et al., 2002; Serror & Sonenshein, 1996), ComK activates its own gene by antagonizing the repressor proteins without preventing their binding (Smits et al., 2007). Because ComK and Rok bind to the minor and major groove of the DNA, respectively, it is postulated that the anti‐repression exerted by ComK is achieved through changes in DNA topology (Smits et al., 2007). Other possibilities might include interference with the DNA bridging behavior of Rok without preventing Rok binding or an as‐yet‐unknown physical interaction between Rok and ComK.

The common denominator in these examples is the unusually stable DNA‐Rok‐DNA bridged complex. The presence of protein partners or the addition or removal of PTMs can potentially connect Rok to various cellular processes such as replication, natural competence and sporulation thereby changing gene expression accordingly (Figure 1c) (Schultz et al., 2009). Rok itself might, therefore, only be indirectly responsive to external cues. Further research on Rok functionality will need to take mechanisms via partner proteins into account.

## 5 IS Rok AN H‐NS‐LIKE PROTEIN?

Dual functionality as genome organizers and gene regulators is one of the characteristics of the H‐NS‐like protein family (Qin et al., 2019). Based on its functional and structural properties, Rok was proposed to be a member of this family (Qin et al., 2019; Smits & Grossman, 2010) which also includes MvaT from *Pseudomonas* sp. and Lsr2 from *Mycobacteria* sp. (Gordon et al., 2008; Tendeng et al., 2003). Structurally, these proteins share an N‐terminal multimerization domain and a C‐terminal DNA binding domain connected by a flexible linker. All these proteins exist (at least) as dimers in solution and they can form DNA–DNA bridges in vitro (Arold et al., 2010; Chen et al., 2008; Dame et al., 2000, 2005, 2006; Ding et al., 2015; Duan et al., 2018; Erkelens et al., 2022; Gordon et al., 2011; Summers et al., 2012; Suzuki‐Minakuchi et al., 2016). Lastly, they all bind the DNA in the minor groove and have a general preference for AT‐rich DNA, especially TpA steps (Ding et al., 2015; Duan et al., 2018, 2021; Gordon et al., 2011; Wiechert et al., 2020). However, in some aspects Rok behaves differently from other H‐NS like proteins. For example, Rok cannot complement *hns* ^−^ phenotypes and so far for Rok a lateral DNA binding state leading to nucleoprotein filament formation has not been observed (Erkelens et al., 2022). This has been attributed to the difference in charge distribution between Rok and other H‐NS‐like proteins (Qin et al., 2019). An important question remains whether Rok should be classified as H‐NS‐like protein, whether an atypical one, or not. The definition of what an H‐NS‐like protein is becomes increasingly pressing as multiple proteins from different bacterial species, that all lack clear sequence homology to H‐NS, have been proposed to be H‐NS‐like proteins in recent years (Baglivo et al., 2018; Barton et al., 2023; Lourenço et al., 2020). Structure prediction programs such as Alphafold2 (Evans et al., 2021; Jumper et al., 2021) could help to identify new potential H‐NS‐like proteins by predicting the presence of a multimerization domain and a DNA binding domain.

A recently proposed new H‐NS‐like protein family member is MucR from the MucR/Ros family of proteins in α‐proteobacteria such as *Brucella* sp. (Baglivo et al., 2018). MucR has a functional role in the regulation of genes important for host infection and virulence (Barton et al., 2023; Caswell et al., 2013; Jiao et al., 2022; Mirabella et al., 2013). Recently, using Hi‐C, it was shown that MucR affects the structure of the *Brucella abortus* nucleoid by preventing short‐ranged contacts along the chromosome (Barton et al., 2023). Structurally, the C‐terminal region of MucR consists of a zinc‐finger domain that is responsible for DNA binding (Palmieri et al., 2013; Russo et al., 2010). The DNA binding preferences of MucR resemble H‐NS as MucR favors AT‐rich DNA with TpA steps and binds in the minor groove (Baglivo et al., 2017). MucR can form higher‐order oligomers via its N‐terminal region (Baglivo et al., 2017). In contrast to the H‐NS dimers, MucR forms decamers in solution (Pirone et al., 2018). The N‐terminal domain was also shown to be necessary for MucR to form DNA bridges and the decamer complex constitutes therefore most likely the functional unit of MucR (Shi et al., 2022). Complementation experiments in *Sinorhizobium fredii* with chimeric proteins show that both functional domains of H‐NS and the N‐terminal domain of Lsr2 can replace those of MucR (Shi et al., 2022). Other studies showed that complementation could also be achieved with the native proteins in both *B. abortus* and *E. coli* (Barton et al., 2023; Shi et al., 2022).

Another NAP that might be an H‐NS‐like protein is GapR from *Caulobacter crescentus* and related α‐proteobacteria (Ricci et al., 2016). The N‐terminal region of GapR shares 61% sequence similarity with H‐NS and a second α‐helix in GapR shares 67% sequence similarity with the C‐terminal DNA binding domain of H‐NS (Lourenço et al., 2020). Complementation experiments with chimeric proteins show that those domains indeed exhibit a similar function, but GapR cannot fully complement the absence of *hns* in *E. coli* (Lourenço et al., 2020). This might be explained by the different protein‐DNA complex that GapR forms compared to H‐NS. GapR forms a tetramer in solution and the protein encircles the DNA like a clamp (Huang et al., 2020; Lourenço et al., 2020; Tarry et al., 2019). Using atomic force microscopy (AFM), GapR was found to stimulate the formation of cross‐overs in DNA, suggestive of DNA bridging observed for H‐NS‐like proteins (Lourenço et al., 2020). It is, however, unclear if this is mediated by individual tetramers or that higher‐order structures are formed. In accordance with its function in chromosome segregation during DNA replication, GapR associates specifically with overtwisted DNA, but also has a binding preference for AT‐rich DNA (Guo et al., 2018). A role in genome organization has been implied for GapR, and global gene expression is affected in its absence (Arias‐Cartin et al., 2017). However, changes in gene expression are most likely an indirect effect as they do not correlate with GapR binding (Guo et al., 2018).

*E. coli* H‐NS has structural and functional features that enable it to play a dual role in genome organization and transcription regulation. As H‐NS is not conserved outside the γ‐proteobacteria, proteins with similar characteristics as described above are expected in other bacteria. In Table 1, we have summarized the functional and structural characteristics of the (proposed) H‐NS‐like proteins. At the structural level, this means a multimerization and a DNA binding domain (Qin et al., 2019). DNA bridging ability has been found for most of the H‐NS‐like proteins, at least in vitro (Chen et al., 2008; Dame et al., 2005; Erkelens et al., 2022). Also, they (nearly) all bind at the minor groove of the DNA (Baglivo et al., 2017; Ding et al., 2015; Duan et al., 2018, 2021; Gordon et al., 2011; Guo et al., 2018; Wiechert et al., 2020). At a functional level, it has been demonstrated that nearly all of these proteins are global transcription regulators (Albano et al., 2005; Castang et al., 2008; Castang & Dove, 2010; Colangeli et al., 2007; Gordon et al., 2010; Mirabella et al., 2013), while a function in genome organization (both global and local) is not always clear yet. A characteristic of H‐NS is its ability to respond to physico‐chemical cues both in vitro and in vivo (Liu et al., 2010; van der Valk et al., 2017). While this is mostly conserved in MvaT and Lsr2, at least in vitro (Qin et al., 2020; Qu et al., 2013; Winardhi et al., 2012), Rok is the notable exception. For the newly proposed H‐NS‐like proteins MucR and GapR, this has not been tested yet. Classically, complementation assays have been used to show if a protein is H‐NS‐like. MvaT, Lsr2 and MucR can indeed replace H‐NS in *E. coli* (Gordon et al., 2008; Tendeng et al., 2003). Again, Rok is an exception. Rok cannot replace H‐NS at the well‐studied *bgl* operon and the growth defect caused by the lack of H‐NS in *E. coli* growth is even aggravated when Rok is expressed (Erkelens et al., 2022). For GapR only chimeric proteins that are part GapR and part H‐NS can complement H‐NS, but not the full‐length protein (Lourenço et al., 2020). However, the high sequence similarities between certain regions of GapR and H‐NS are suggestive of a shared evolutionary history. Therefore, it is unclear if a lack of *hns* complementation is merely a reflection of incompatibilities between bacterial species instead of the protein not being H‐NS‐like.

**TABLE 1:** Characteristics of (proposed) H‐NS‐like proteins.

		H‐NS	MvaT	Lsr2	Rok	MucR	GapR
Bacteria		*Enterbacteriaceae* (−)	*Pseudomonas* sp. (−)	*Actinomycetes* (+)	*Bacillus* sp. (+)	*α‐proteobacteria* (−)	*Caulobacter* sp. (−)
Sequence homology with H‐NS (%)		–	36.4	20.6	30	21.8	32.4
# molecules per cell (*Protein or **RNA)		4000–18,000*	2000–16,000**	350–1400 RPKM**	1000–3000*	?	?
Protomer size		Dimer	Dimer	Dimer	Unknown	Decamer	Tetramer
Genome organizer (in vivo)	Global	**?**	**?**	**?**	**V**	**V**	**±**
	Local	**V**	**?**	**?**	**?**	**?**	**?**
Global transcriptional regulator		**V**	**V**	**V**	**V**	**V**	**±**
Multimerization domain		**V**	**V**	**V**	**V**	**V**	**V**
DNA binding domain		**V**	**V**	**V**	**V**	**V**	**V**
Nucleofilament formation	In vitro	**V**	**V**	**V**	**?**	**?**	**?**
	In vivo	**V**	**?**	**?**	**?**	**?**	**?**
DNA bridging	In vitro	**V**	**V**	**V**	**V**	**V**	**±**
	In vivo	**V**	**?**	**?**	**±**	**?**	**?**
Reacts to physico‐chemical cues	In vitro	**V**	**V**	**±**	**X**	**?**	**?**
	In vivo	**V**	**?**	**?**	**?**	**?**	**?**
Complementation of *hns* ^−^ phenotype		**V**	**V**	**V**	**X**	**V**	**±**

*Note*: Gram‐negative and Gram‐positive bacterial species are indicated with (−) and (+), respectively. Green V: this has been shown for this protein. Red X: it has been shown that the protein does not have this characteristic. Yellow ±: it has been shown partially or has been implied/suggested. Gray ?: this is currently unknown. The sequence similarity was determined using EMBOSS Needle with the following NCBI accession numbers: H‐NS: NP_415753.1, MvaT: NP_253005.1, Lsr2: NP_218114.1, Rok: NP_389307.1, MucR: WP_002963720.1, GapR: WP_010921151.1, RPKM: reads per kilobase of transcript per million mapped reads. *The amount of protein molecules per cell was taken from Amemiya et al. (2021) and Smits & Grossman (2010). **For MvaT the RNA levels were estimated from www.pseudomonas.org and for Lsr2 from Wang et al. (2013), where the RNA polymerase gene *rpoB* shows levels between 400 and 800 RPKM.

In summary, this table gives an idea of the shared characteristics between the different proteins and provides a framework in deciding whether to call a protein an H‐NS‐like protein.

## 6 CONCLUSION AND PERSPECTIVES

The *B. subtilis* genome is organized at multiple hierarchical levels, orchestrated in large part by the actions of architectural chromatin proteins, such as BsSMC, HBsu and Rok (Figure 1). This organization impacts genomic transactions such as replication and transcription. Rok has a dual role as genome organizer and gene regulator. Here, we summarized studies focusing on either one of these two functions of Rok and tried to establish meaningful connections. Although studies that address both sides of the coin in an integrated manner are lacking, we propose that the stable DNA–DNA bridges formed by Rok are responsible for gene repression (Figure 1c). Partner proteins such as sRok and DnaA can affect the bridging activity of Rok, which in turn may modulate its regulatory role. This dual role and the interplay between structure and function also holds for H‐NS. H‐NS regulates gene expression by chromatin‐remodeling, effectively coupling chromosome structure with transcription (Rashid et al., 2023). Rok likely exerts a similar interplay between these two processes. As Rok is involved in processes like competence and sporulation (Schultz et al., 2009), differences in genome structure are expected in addition to transcriptional changes. In Table 1 we propose a list of characteristics to determine if a protein can be considered H‐NS‐like. This table also shows that several of these characteristics of the recently proposed H‐NS‐like proteins have not been studied yet, underlining how active this field of research is with many open questions and potentially new proteins to be identified. Based on lessons learned so far, further research might explicitly take the coupling between chromosome structure and gene regulation into account. This requires an integrated combination of in vivo approaches like 3C‐based techniques, RNA‐seq, ideally at the single‐cell level, and super‐resolution microscopy with (single‐molecule) in vitro experiments.

## AUTHOR CONTRIBUTIONS

**Remus T. Dame:** Conceptualization; investigation; writing – review and editing; supervision; funding acquisition. **Amanda M. Erkelens:** Conceptualization; investigation; writing – original draft; writing – review and editing. **Bert van Erp:** Conceptualization; investigation; writing – original draft; writing – review and editing. **Wilfried J. J. Meijer:** Writing – review and editing.

## CONFLICT OF INTEREST STATEMENT

The authors declare no conflict of interests.

## ETHICS STATEMENT

All authors agree with the contents of this article.

## Data Availability

Data sharing not applicable to this article as no datasets were generated or analysed during the current study.

## References

[mmi15250-bib-0001] Albano, M. , Smits, W.K. , Ho, L.T.Y. , Kraigher, B. , Mandic‐Mulec, I. , Kuipers, O.P. et al. (2005) The Rok protein of *Bacillus subtilis* represses genes for cell surface and extracellular functions. Journal of Bacteriology, 187(6), 2010–2019. Available from: 10.1128/JB.187.6.2010-2019.2005 15743949 PMC1064057

[mmi15250-bib-0002] Al‐Husini, N. , Tomares, D.T. , Bitar, O. , Childers, W.S. & Schrader, J.M. (2018) α‐Proteobacterial RNA Degradosomes assemble liquid‐liquid phase‐separated RNP bodies. Molecular Cell, 71(6), 1027–1039.e14. Available from: 10.1016/J.MOLCEL.2018.08.003 30197298 PMC6151146

[mmi15250-bib-0003] Ali Azam, T. , Iwata, A. , Nishimura, A. , Ueda, S. & Ishihama, A. (1999) Growth phase‐dependent variation in protein composition of the *Escherichia coli* nucleoid. Journal of Bacteriology, 181(20), 6361–6370. Available from: 10.1128/JB.181.20.6361-6370.1999 10515926 PMC103771

[mmi15250-bib-0004] Alipour, E. & Marko, J.F. (2012) Self‐organization of domain structures by DNA‐loop‐extruding enzymes. Nucleic Acids Research, 40(22), 11202–11212. Available from: 10.1093/NAR/GKS925 23074191 PMC3526278

[mmi15250-bib-0005] Amemiya, H.M. , Schroeder, J. & Freddolino, P.L. (2021) Nucleoid‐associated proteins shape chromatin structure and transcriptional regulation across the bacterial kingdom. Transcription, 12(4), 182–218. Available from: 10.1080/21541264.2021.1973865 34499567 PMC8632127

[mmi15250-bib-0006] Amit, R. , Oppenheim, A.B. & Stavans, J. (2003) Increased bending rigidity of single DNA molecules by H‐NS, a temperature and osmolarity sensor. Biophysical Journal, 84(4), 2467–2473. Available from: 10.1016/S0006-3495(03)75051-6 12668454 PMC1302812

[mmi15250-bib-0007] Anderson, D.E. , Losada, A. , Erickson, H.P. & Hirano, T. (2002) Condensin and cohesin display different arm conformations with characteristic hinge angles. The Journal of Cell Biology, 156(3), 419–424. Available from: 10.1083/JCB.200111002 11815634 PMC2173330

[mmi15250-bib-0008] Antelmann, H. , Engelmann, S. , Schmid, R. , Sorokin, A. , Lapidus, A. & Hecker, M. (1997) Expression of a stress‐ and starvation‐induced *dps/pexB*‐homologous gene is controlled by the alternative sigma factor *sigmaB* in *Bacillus subtilis* . Journal of Bacteriology, 179(23), 7251–7256. Available from: 10.1128/jb.179.23.7251-7256.1997 9393687 PMC179673

[mmi15250-bib-0009] Arias‐Cartin, R. , Dobihal, G.S. , Campos, M. , Surovtsev, I.V. , Parry, B. & Jacobs‐Wagner, C. (2017) Replication fork passage drives asymmetric dynamics of a critical nucleoid‐associated protein in *Caulobacter* . The EMBO Journal, 36(3), 301–318. Available from: 10.15252/embj.201695513 28011580 PMC5286365

[mmi15250-bib-0010] Arold, S.T. , Leonard, P.G. , Parkinson, G.N. & Ladbury, J.E. (2010) H‐NS forms a superhelical protein scaffold for DNA condensation. Proceedings of the National Academy of Sciences, 107(36), 15728–15732. Available from: 10.1073/pnas.1006966107 PMC2936596 20798056

[mmi15250-bib-0011] Atlung, T. , Knudsen, K. , Heerfordt, L. & Brøndsted, L. (1997) Effects of *sigmaS* and the transcriptional activator AppY on induction of the *Escherichia coli< hya* and *cbdAB‐appA* operons in response to carbon and phosphate starvation. Journal of Bacteriology, 179(7), 2141–2146. Available from: 10.1128/jb.179.7.2141-2146.1997 9079897 PMC178948

[mmi15250-bib-0012] Azaldegui, C.A. , Vecchiarelli, A.G. & Biteen, J.S. (2021) The emergence of phase separation as an organizing principle in bacteria. Biophysical Journal, 120(7), 1123–1138. Available from: 10.1016/J.BPJ.2020.09.023 33186556 PMC8059088

[mmi15250-bib-0013] Azam, T.A. & Ishihama, A. (1999) Twelve species of the nucleoid‐associated protein from *Escherichia coli* . Journal of Biological Chemistry, 274(46), 33105–33113. Available from: 10.1074/jbc.274.46.33105 10551881

[mmi15250-bib-0014] Baglivo, I. , Pirone, L. , Malgieri, G. , Fattorusso, R. , Roop, R.M. , Pedone, E.M. et al. (2018) MucR binds multiple target sites in the promoter of its own gene and is a heat‐stable protein: is MucR a H‐NS‐like protein? FEBS Open Bio, 8(4), 711–718. Available from: 10.1002/2211-5463.12411 PMC5881533 29632823

[mmi15250-bib-0015] Baglivo, I. , Pirone, L. , Pedone, E.M. , Pitzer, J.E. , Muscariello, L. , Marino, M.M. et al. (2017) Ml proteins from *Mesorhizobium loti* and MucR from *Brucella abortus*: an AT‐rich core DNA‐target site and oligomerization ability. Scientific Reports, 7(1), 15805. Available from: 10.1038/s41598-017-16127-5 29150637 PMC5693944

[mmi15250-bib-0016] Barton, I.S. , Ren, Z. , Cribb, C.B. , Pitzer, J.E. , Baglivo, I. , Martin, D.W. et al. (2023) *Brucella* MucR acts as an H‐NS‐like protein to silence virulence genes and structure the nucleoid. MBio, 14(6), e02201‐23. Available from: 10.1128/mbio.02201-23 37847580 PMC10746212

[mmi15250-bib-0017] Bock, F.P. , Liu, H.W. , Anchimiuk, A. , Diebold‐Durand, M.‐L. & Gruber, S. (2022) A joint‐ParB interface promotes Smc DNA recruitment. Cell Reports, 40(9), 111273. Available from: 10.1016/j.celrep.2022.111273 36044845 PMC9449133

[mmi15250-bib-0018] Boeynaems, S. , Alberti, S. , Fawzi, N.L. , Mittag, T. , Polymenidou, M. , Rousseau, F. et al. (2018) Protein phase separation: a new phase in cell biology. Trends in Cell Biology, 28(6), 420–435. Available from: 10.1016/J.TCB.2018.02.004 29602697 PMC6034118

[mmi15250-bib-0019] Boija, A. , Klein, I.A. , Sabari, B.R. , Dall'Agnese, A. , Coffey, E.L. , Zamudio, A.V. et al. (2018) Transcription factors activate genes through the phase‐separation capacity of their activation domains. Cell, 175(7), 1842–1855.e16. Available from: 10.1016/J.CELL.2018.10.042 30449618 PMC6295254

[mmi15250-bib-0020] Boudreau, B.A. , Hron, D.R. , Qin, L. , van der Valk, R.A. , Kotlajich, M.V. , Dame, R.T. et al. (2018) StpA and Hha stimulate pausing by RNA polymerase by promoting DNA–DNA bridging of H‐NS filaments. Nucleic Acids Research, 46(11), 5525–5546. Available from: 10.1093/nar/gky265 29718386 PMC6009659

[mmi15250-bib-0021] Brandão, H.B. , Ren, Z. , Karaboja, X. , Mirny, L.A. & Wang, X. (2021) DNA‐loop‐extruding SMC complexes can traverse one another *in vivo* . Nature Structural & Molecular Biology, 28(8), 642–651. Available from: 10.1038/s41594-021-00626-1 PMC8878250 34312537

[mmi15250-bib-0022] Carabetta, V.J. , Greco, T.M. , Cristea, I.M. & Dubnau, D. (2019) YfmK is an N^ε^‐lysine acetyltransferase that directly acetylates the histone‐like protein HBsu in *Bacillus subtilis* . Proceedings of the National Academy of Sciences of the United States of America, 116(9), 3752–3757. Available from: 10.1073/pnas.1815511116 30808761 PMC6397556

[mmi15250-bib-0023] Carabetta, V.J. , Greco, T.M. , Tanner, A.W. , Cristea, I.M. & Dubnau, D. (2016) Temporal regulation of the *Bacillus subtilis* acetylome and evidence for a role of MreB acetylation in cell wall growth. mSystems, 1(3), e00005‐16. Available from: 10.1128/mSystems.00005-16 27376153 PMC4927096

[mmi15250-bib-0024] Castang, S. & Dove, S.L. (2010) High‐order oligomerization is required for the function of the H‐NS family member MvaT in *Pseudomonas aeruginosa* . Molecular Microbiology, 78(4), 916–931. Available from: 10.1111/j.1365-2958.2010.07378.x 20815825 PMC2978250

[mmi15250-bib-0025] Castang, S. , McManus, H.R. , Turner, K.H. & Dove, S.L. (2008) H‐NS family members function coordinately in an opportunistic pathogen. Proceedings of the National Academy of Sciences of the United States of America, 105(48), 18947–18952. Available from: 10.1073/pnas.0808215105 19028873 PMC2596223

[mmi15250-bib-0026] Caswell, C.C. , Elhassanny, A.E.M. , Planchin, E.E. , Roux, C.M. , Weeks‐Gorospe, J.N. , Ficht, T.A. et al. (2013) Diverse genetic regulon of the virulence‐associated transcriptional regulator MucR in *Brucella abortus 2308* . Infection and Immunity, 81(4), 1040–1051. Available from: 10.1128/IAI.01097-12 23319565 PMC3639602

[mmi15250-bib-0027] Chen, J.M. , Ren, H. , Shaw, J.E. , Wang, Y.J. , Li, M. , Leung, A.S. et al. (2008) Lsr2 of *Mycobacterium tuberculosis* is a DNA‐bridging protein. Nucleic Acids Research, 36(7), 2123–2135. Available from: 10.1093/nar/gkm1162 18187505 PMC2367712

[mmi15250-bib-0028] Chodavarapu, S. , Felczak, M.M. , Yaniv, J.R. & Kaguni, J.M. (2008) *Escherichia coli* DnaA interacts with HU in initiation at the *E. coli* replication origin. Molecular Microbiology, 67(4), 781–792. Available from: 10.1111/j.1365-2958.2007.06094.x 18179598

[mmi15250-bib-0029] Choi, J. , Schmukler, M. & Groisman, E.A. (2022) Degradation of gene silencer is essential for expression of foreign genes and bacterial colonization of the mammalian gut. Proceedings of the National Academy of Sciences, 119(40), e2210239119. Available from: 10.1073/pnas.2210239119 PMC9546599 36161931

[mmi15250-bib-0030] Cobbe, N. & Heck, M.M.S. (2004) The evolution of SMC proteins: phylogenetic analysis and structural implications. Molecular Biology and Evolution, 21(2), 332–347. Available from: 10.1093/molbev/msh023 14660695

[mmi15250-bib-0031] Cohan, M.C. & Pappu, R.V. (2020) Making the case for disordered proteins and biomolecular condensates in bacteria. Trends in Biochemical Sciences, 45(8), 668–680. Available from: 10.1016/j.tibs.2020.04.011 32456986

[mmi15250-bib-0032] Colangeli, R. , Helb, D. , Vilchèze, C. , Hazbón, M.H. , Lee, C.‐G. , Safi, H. et al. (2007) Transcriptional regulation of multi‐drug tolerance and antibiotic‐induced responses by the histone‐like protein Lsr2 in *M. Tuberculosis* . PLoS Pathogens, 3(6), e87. Available from: 10.1371/journal.ppat.0030087 17590082 PMC1894825

[mmi15250-bib-0033] Crémazy, F.G. , Rashid, F.Z.M. , Haycocks, J.R. , Lamberte, L.E. , Grainger, D.C. & Dame, R.T. (2018) Determination of the 3D genome organization of bacteria using hi‐C. Methods in Molecular Biology (Clifton, N.J.), 1837, 3–18. Available from: 10.1007/978-1-4939-8675-0_1 30109602

[mmi15250-bib-0034] da Costa‐Nunes, J.A. & Noordermeer, D. (2023) TADs: dynamic structures to create stable regulatory functions. Current Opinion in Structural Biology, 81, 102622. Available from: 10.1016/J.SBI.2023.102622 37302180

[mmi15250-bib-0035] Dame, R.T. (2005) The role of nucleoid‐associated proteins in the organization and compaction of bacterial chromatin. Molecular Microbiology, 56(4), 858–870. Available from: 10.1111/j.1365-2958.2005.04598.x 15853876

[mmi15250-bib-0036] Dame, R.T. , Luijsterburg, M.S. , Krin, E. , Bertin, P.N. , Wagner, R. & Wuite, G.J.L. (2005) DNA bridging: a property shared among H‐NS‐like proteins. Journal of Bacteriology, 187(5), 1845–1848. Available from: 10.1128/JB.187.5.1845-1848.2005 15716456 PMC1064010

[mmi15250-bib-0037] Dame, R.T. , Noom, M.C. & Wuite, G.J.L. (2006) Bacterial chromatin organization by H‐NS protein unravelled using dual DNA manipulation. Nature, 444(7117), 387–390. Available from: 10.1038/nature05283 17108966

[mmi15250-bib-0038] Dame, R.T. , Rashid, F.‐Z.M. & Grainger, D.C. (2020) Chromosome organization in bacteria: mechanistic insights into genome structure and function. Nature Reviews Genetics, 21(4), 227–242. Available from: 10.1038/s41576-019-0185-4 31767998

[mmi15250-bib-0039] Dame, R.T. , Wyman, C. & Goosen, N. (2000) H‐NS mediated compaction of DNA visualised by atomic force microscopy. Nucleic Acids Research, 28(18), 3504–3510. Available from: 10.1093/nar/28.18.3504 10982869 PMC110753

[mmi15250-bib-0040] Dame, R.T. , Wyman, C. , Wurm, R. , Wagner, R. & Goosen, N. (2002) Structural basis for H‐NS‐mediated trapping of RNA polymerase in the open initiation complex at the *rrnB* P1. Journal of Biological Chemistry, 277(3), 2146–2150. Available from: 10.1074/jbc.C100603200 11714691

[mmi15250-bib-0041] Danilova, O. , Reyes‐Lamothe, R. , Pinskaya, M. , Sherratt, D. & Possoz, C. (2007) MukB colocalizes with the *oriC* region and is required for organization of the two *Escherichia coli* chromosome arms into separate cell halves. Molecular Microbiology, 65(6), 1485–1492. Available from: 10.1111/j.1365-2958.2007.05881.x 17824928 PMC2169520

[mmi15250-bib-0042] Dekker, J. , Rippe, K. , Dekker, M. & Kleckner, N. (2002) Capturing chromosome conformation. Science (New York, N.Y.), 295(5558), 1306–1311. Available from: 10.1126/SCIENCE.1067799 11847345

[mmi15250-bib-0043] Denham, E.L. , Piersma, S. , Rinket, M. , Reilman, E. , de Goffau, M.C. & van Dijl, J.M. (2019) Differential expression of a prophage‐encoded glycocin and its immunity protein suggests a mutualistic strategy of a phage and its host. Scientific Reports, 9(1), 2845. Available from: 10.1038/s41598-019-39169-3 30808982 PMC6391423

[mmi15250-bib-0044] Dignon, G.L. , Best, R.B. & Mittal, J. (2020) Biomolecular phase separation: from molecular driving forces to macroscopic properties. Annual Review of Physical Chemistry, 71, 53–75. Available from: 10.1146/ANNUREV-PHYSCHEM-071819-113553 PMC7469089 32312191

[mmi15250-bib-0045] Dilweg, I.W. & Dame, R.T. (2018) Post‐translational modification of nucleoid‐associated proteins: an extra layer of functional modulation in bacteria? Biochemical Society Transactions, 46(5), 1381–1392. Available from: 10.1042/BST20180488 30287510

[mmi15250-bib-0046] Ding, P. , McFarland, K.A. , Jin, S. , Tong, G. , Duan, B. , Yang, A. et al. (2015) A novel AT‐rich DNA recognition mechanism for bacterial xenogeneic silencer MvaT. PLoS Pathogens, 11(6), e1004967. Available from: 10.1371/journal.ppat.1004967 26068099 PMC4466236

[mmi15250-bib-0047] Dixon, J.R. , Selvaraj, S. , Yue, F. , Kim, A. , Li, Y. , Shen, Y. et al. (2012) Topological domains in mammalian genomes identified by analysis of chromatin interactions. Nature, 485(7398), 376–380. Available from: 10.1038/nature11082 22495300 PMC3356448

[mmi15250-bib-0048] Duan, B. , Ding, P. , Hughes, T.R. , Navarre, W.W. , Liu, J. & Xia, B. (2018) How bacterial xenogeneic silencer Rok distinguishes foreign from self DNA in its resident genome. Nucleic Acids Research, 46(19), 10514–10529. Available from: 10.1093/nar/gky836 30252102 PMC6212790

[mmi15250-bib-0049] Duan, B. , Ding, P. , Navarre, W.W. , Liu, J. & Xia, B. (2021) Xenogeneic silencing and bacterial genome evolution: mechanisms for DNA recognition imply multifaceted roles of xenogeneic silencers. Molecular Biology and Evolution, 38(10), 4135. Available from: 10.1093/MOLBEV/MSAB136 34003286 PMC8476142

[mmi15250-bib-0050] Dugar, G. , Hofmann, A. , Heermann, D.W. & Hamoen, L.W. (2022) A chromosomal loop anchor mediates bacterial genome organization. Nature Genetics, 54(2), 194–201. Available from: 10.1038/s41588-021-00988-8 35075232

[mmi15250-bib-0051] Elsholz, A.K.W. , Turgay, K. , Michalik, S. , Hessling, B. , Gronau, K. , Oertel, D. et al. (2012) Global impact of protein arginine phosphorylation on the physiology of *Bacillus subtilis* . Proceedings of the National Academy of Sciences of the United States of America, 109(19), 7451–7456. Available from: 10.1073/pnas.1117483109 22517742 PMC3358850

[mmi15250-bib-0052] Erkelens, A.M. , Qin, L. , van Erp, B. , Miguel‐Arribas, A. , Abia, D. , Keek, H.G.J. et al. (2022) The *B. subtilis* Rok protein is an atypical H‐NS‐like protein irresponsive to physico‐chemical cues. Nucleic Acids Research, 50(21), 12166–12185. Available from: 10.1093/nar/gkac1064 36408910 PMC9757077

[mmi15250-bib-0053] Errington, J. & van der Aart, L.T. (2020) Microbe profile: *Bacillus subtilis*: model organism for cellular development, and industrial workhorse. Microbiology, 166(5), 425–427. Available from: 10.1099/mic.0.000922 32391747 PMC7376258

[mmi15250-bib-0054] Evans, R. , O'Neill, M. , Pritzel, A. , Antropova, N. , Senior, A. , Green, T. et al. (2021) Protein complex prediction with AlphaFold‐Multimer. BioRxiv 10.1101/2021.10.04.463034

[mmi15250-bib-0055] Feklístov, A. , Sharon, B.D. , Darst, S.A. & Gross, C.A. (2014) Bacterial sigma factors: a historical, structural, and genomic perspective. Annual Review of Microbiology, 68(1), 357–376. Available from: 10.1146/annurev-micro-092412-155737 25002089

[mmi15250-bib-0056] Feric, M. & Misteli, T. (2021) Phase separation in genome organization across evolution. Trends in Cell Biology, 31(8), 671–685. Available from: 10.1016/J.TCB.2021.03.001 33771451 PMC8286288

[mmi15250-bib-0057] Forrest, D. , Warman, E.A. , Erkelens, A.M. , Dame, R.T. & Grainger, D.C. (2022) Xenogeneic silencing strategies in bacteria are dictated by RNA polymerase promiscuity. Nature Communications, 13(1), 1149. Available from: 10.1038/s41467-022-28747-1 PMC8894471 35241653

[mmi15250-bib-0058] Fudenberg, G. , Imakaev, M. , Lu, C. , Goloborodko, A. , Abdennur, N. & Mirny, L.A. (2016) Formation of chromosomal domains by loop extrusion. Cell Reports, 15(9), 2038–2049. Available from: 10.1016/J.CELREP.2016.04.085 27210764 PMC4889513

[mmi15250-bib-0059] Galperin, M.Y. , Yutin, N. , Wolf, Y.I. , Alvarez, R.V. & Koonin, E.V. (2022) Conservation and evolution of the sporulation gene set in diverse members of the *Firmicutes* . Journal of Bacteriology, 204(6), e0007922. Available from: 10.1128/JB.00079-22 35638784 PMC9210971

[mmi15250-bib-0060] Gogou, C. , Japaridze, A. & Dekker, C. (2021) Mechanisms for chromosome segregation in bacteria. Frontiers in Microbiology, 12, 685687. Available from: 10.3389/FMICB.2021.685687 34220773 PMC8242196

[mmi15250-bib-0061] Göransson, M. , Sondén, B. , Nilsson, P. , Dagberg, B. , Foreman, K. , Emanuelsson, K. et al. (1990) Transcriptional silencing and thermoregulation of gene expression in *Escherichia coli* . Nature, 344(6267), 682–685. Available from: 10.1038/344682a0 1691451

[mmi15250-bib-0062] Gordon, B.R.G. , Imperial, R. , Wang, L. , Navarre, W.W. & Liu, J. (2008) Lsr2 of *Mycobacterium* represents a novel class of H‐NS‐like proteins. Journal of Bacteriology, 190(21), 7052–7059. Available from: 10.1128/JB.00733-08 18776007 PMC2580683

[mmi15250-bib-0063] Gordon, B.R.G. , Li, Y. , Cote, A. , Weirauch, M.T. , Ding, P. , Hughes, T.R. et al. (2011) Structural basis for recognition of AT‐rich DNA by unrelated xenogeneic silencing proteins. Proceedings of the National Academy of Sciences of the United States of America, 108(26), 10690–10695. Available from: 10.1073/pnas.1102544108 21673140 PMC3127928

[mmi15250-bib-0064] Gordon, B.R.G. , Li, Y. , Wang, L. , Sintsova, A. , van Bakel, H. , Tian, S. et al. (2010) Lsr2 is a nucleoid‐associated protein that targets AT‐rich sequences and virulence genes in *Mycobacterium tuberculosis* . Proceedings of the National Academy of Sciences of the United States of America, 107(11), 5154–5159. Available from: 10.1073/pnas.0913551107 20133735 PMC2841939

[mmi15250-bib-0065] Grove, A. (2011) Functional evolution of bacterial histone‐like HU proteins. Current Issues in Molecular Biology, 13(1), 1–12. Available from: 10.21775/cimb.013.001 20484776

[mmi15250-bib-0066] Gruber, S. , Veening, J.‐W. , Bach, J. , Blettinger, M. , Bramkamp, M. & Errington, J. (2014) Interlinked sister chromosomes Arise in the absence of Condensin during fast replication in *B. subtilis* . Current Biology, 24(3), 293–298. Available from: 10.1016/j.cub.2013.12.049 24440399 PMC3919155

[mmi15250-bib-0067] Guilhas, B. , Walter, J.‐C. , Rech, J. , Bouet, J.‐Y. , Le Gall, A. & Nollmann, M. (2020) ATP‐driven separation of liquid phase condensates in bacteria. Molecular Cell, 79, 293–303.e4. Available from: 10.1016/j.molcel.2020.06.034 32679076

[mmi15250-bib-0068] Guo, M.S. , Haakonsen, D.L. , Zeng, W. , Schumacher, M.A. & Laub, M.T. (2018) A bacterial chromosome structuring protein binds overtwisted DNA to stimulate type II topoisomerases and enable DNA replication. Cell, 175(2), 583–597. Available from: 10.1016/j.cell.2018.08.029 30220456 PMC6173638

[mmi15250-bib-0069] Gupta, A. , Joshi, A. , Arora, K. , Mukhopadhyay, S. & Guptasarma, P. (2023) The bacterial nucleoid‐associated proteins, HU and Dps, condense DNA into context‐dependent biphasic or multiphasic complex coacervates. Journal of Biological Chemistry, 299(5), 104637. Available from: 10.1016/j.jbc.2023.104637 36963493 PMC10141540

[mmi15250-bib-0070] Haering, C.H. , Schoffnegger, D. , Nishino, T. , Helmhart, W. , Nasmyth, K. & Löwe, J. (2004) Structure and stability of Cohesin's Smc1‐Kleisin interaction. Molecular Cell, 15(6), 951–964. Available from: 10.1016/J.MOLCEL.2004.08.030 15383284

[mmi15250-bib-0071] Hammel, M. , Amlanjyoti, D. , Reyes, F.E. , Chen, J.H. , Parpana, R. , Tang, H.Y.H. et al. (2016) HU multimerization shift controls nucleoid compaction. Science Advances, 2(7), e1600650. Available from: 10.1126/SCIADV.1600650/SUPPL_FILE/1600650_SM.PDF 27482541 PMC4966879

[mmi15250-bib-0072] Hamoen, L.W. , Kausche, D. , Marahiel, M.A. , Sinderen, D. , Venema, G. & Serror, P. (2003) The *Bacillus subtilis* transition state regulator AbrB binds to the ‐35 promoter region of *comK* . FEMS Microbiology Letters, 218(2), 299–304. Available from: 10.1111/j.1574-6968.2003.tb11532.x 12586407

[mmi15250-bib-0073] Hiraga, S. , Niki, H. , Ogura, T. , Ichinose, C. , Mori, H. , Ezaki, B. et al. (1989) Chromosome partitioning in *Escherichia coli*: novel mutants producing anucleate cells. Journal of Bacteriology, 171(3), 1496–1505. Available from: 10.1128/jb.171.3.1496-1505.1989 2646284 PMC209772

[mmi15250-bib-0074] Hoa, T.T. , Tortosa, P. , Albano, M. & Dubnau, D. (2002) Rok (YkuW) regulates genetic competence in *Bacillus subtilis* by directly repressing *comK* . Molecular Microbiology, 43(1), 15–26. Available from: 10.1046/j.1365-2958.2002.02727.x 11849533

[mmi15250-bib-0075] Hommais, F. , Krin, E. , Laurent‐Winter, C. , Soutourina, O. , Malpertuy, A. , Le Caer, J.P. et al. (2001) Large‐scale monitoring of pleiotropic regulation of gene expression by the prokaryotic nucleoid‐associated protein, H‐NS. Molecular Microbiology, 40(1), 20–36 http://www.ncbi.nlm.nih.gov/pubmed/11298273 11298273 10.1046/j.1365-2958.2001.02358.x

[mmi15250-bib-0076] Hsieh, T.H.S. , Weiner, A. , Lajoie, B. , Dekker, J. , Friedman, N. & Rando, O.J. (2015) Mapping nucleosome resolution chromosome folding in yeast by micro‐C. Cell, 162(1), 108–119. Available from: 10.1016/J.CELL.2015.05.048 26119342 PMC4509605

[mmi15250-bib-0077] Huang, Q. , Duan, B. , Dong, X. , Fan, S. & Xia, B. (2020) GapR binds DNA through dynamic opening of its tetrameric interface. Nucleic Acids Research, 48(16), 9372–9386. Available from: 10.1093/NAR/GKAA644 32756896 PMC7498317

[mmi15250-bib-0078] Ishikawa, S. , Ogura, Y. , Yoshimura, M. , Okumura, H. , Cho, E. , Kawai, Y. et al. (2007) Distribution of stable DnaA‐binding sites on the *Bacillus subtilis* genome detected using a modified ChIP‐chip method. DNA Research, 14(4), 155–168. Available from: 10.1093/dnares/dsm017 17932079 PMC2533591

[mmi15250-bib-0079] Jalal, A.S.B. & Le, T.B.K. (2020) Bacterial chromosome segregation by the ParABS system. Open Biology, 10(6), 200097. Available from: 10.1098/RSOB.200097 32543349 PMC7333895

[mmi15250-bib-0080] Janissen, R. , Arens, M.M.A. , Vtyurina, N.N. , Rivai, Z. , Sunday, N.D. , Eslami‐Mossallam, B. et al. (2018) Global DNA compaction in stationary‐phase bacteria does not affect transcription. Cell, 174(5), 1188–1199.e14. Available from: 10.1016/J.CELL.2018.06.049 30057118 PMC6108918

[mmi15250-bib-0081] Jiao, J. , Zhang, B. , Li, M.‐L. , Zhang, Z. & Tian, C.‐F. (2022) The zinc‐finger bearing xenogeneic silencer MucR in α‐proteobacteria balances adaptation and regulatory integrity. The ISME Journal, 16(3), 738–749. Available from: 10.1038/s41396-021-01118-2 34584215 PMC8857273

[mmi15250-bib-0082] Johansson, J. , Eriksson, S. , Sondén, B. , Wai, S.N. & Uhlin, B.E. (2001) Heteromeric interactions among nucleoid‐associated bacterial proteins: localization of StpA‐stabilizing regions in H‐NS of *Escherichia coli* . Journal of Bacteriology, 183(7), 2343–2347. Available from: 10.1128/JB.183.7.2343-2347.2001 11244076 PMC95143

[mmi15250-bib-0083] Johansson, J. & Uhlin, B.E. (1999) Differential protease‐mediated turnover of H‐NS and StpA revealed by a mutation altering protein stability and stationary‐phase survival of *Escherichia coli* . Proceedings of the National Academy of Sciences of the United States of America, 96(19), 10776–10781. Available from: 10.1073/pnas.96.19.10776 10485902 PMC17959

[mmi15250-bib-0084] Joyeux, M. (2018) A segregative phase separation scenario of the formation of the bacterial nucleoid. Soft Matter, 14(36), 7368–7381. Available from: 10.1039/C8SM01205A 30204212

[mmi15250-bib-0085] Jumper, J. , Evans, R. , Pritzel, A. , Green, T. , Figurnov, M. , Ronneberger, O. et al. (2021) Highly accurate protein structure prediction with AlphaFold. Nature, 596(7873), 583–589. Available from: 10.1038/s41586-021-03819-2 34265844 PMC8371605

[mmi15250-bib-0086] Kaguni, J.M. (2006) DnaA: controlling the initiation of bacterial DNA replication and more. Annual Review of Microbiology, 60(1), 351–371. Available from: 10.1146/annurev.micro.60.080805.142111 16753031

[mmi15250-bib-0087] Karaboja, X. & Wang, X. (2022) HBsu is required for the initiation of DNA replication in *Bacillus subtilis* . Journal of Bacteriology, 204(8), e0011922. Available from: 10.1128/jb.00119-22 35546541 PMC9380562

[mmi15250-bib-0088] Kim, E. , Barth, R. & Dekker, C. (2023) Looping the genome with SMC complexes. Annual Review of Biochemistry, 92(1), 15–41. Available from: 10.1146/annurev-biochem-032620-110506 37137166

[mmi15250-bib-0089] Kim, J. (2004) Fundamental structural units of the *Escherichia coli* nucleoid revealed by atomic force microscopy. Nucleic Acids Research, 32(6), 1982–1992. Available from: 10.1093/nar/gkh512 15060178 PMC390363

[mmi15250-bib-0090] Köhler, P. & Marahiel, M.A. (1997) Association of the histone‐like protein HBsu with the nucleoid of *Bacillus subtilis* . Journal of Bacteriology, 179(6), 2060–2064. Available from: 10.1128/jb.179.6.2060-2064.1997 9068655 PMC178933

[mmi15250-bib-0091] Kovács, A.T. & Kuipers, O.P. (2011) Rok regulates *yuaB* expression during architecturally complex Colony development of *Bacillus subtilis* 168. Journal of Bacteriology, 193(4), 998–1002. Available from: 10.1128/JB.01170-10 21097620 PMC3028688

[mmi15250-bib-0092] Kunst, F. , Ogasawara, N. , Moszer, I. , Albertini, A.M. , Alloni, G. , Azevedo, V. et al. (1997) The complete genome sequence of the gram‐positive bacterium *Bacillus subtilis* . Nature, 390(6657), 249–256. Available from: 10.1038/36786 9384377

[mmi15250-bib-0093] Ladouceur, A.‐M. , Parmar, B.S. , Biedzinski, S. , Wall, J. , Tope, S.G. , Cohn, D. et al. (2020) Clusters of bacterial RNA polymerase are biomolecular condensates that assemble through liquid–liquid phase separation. Proceedings of the National Academy of Sciences of the United States of America, 117(31), 18540–18549. Available from: 10.1073/pnas.2005019117 32675239 PMC7414142

[mmi15250-bib-0094] Lammens, A. , Schele, A. & Hopfner, K.P. (2004) Structural biochemistry of ATP‐driven dimerization and DNA‐stimulated activation of SMC ATPases. Current Biology, 14(19), 1778–1782. Available from: 10.1016/J.CUB.2004.09.044 15458651

[mmi15250-bib-0095] Le, T.B.K. , Imakaev, M.V. , Mirny, L.A. & Laub, M.T. (2013) High‐resolution mapping of the spatial organization of a bacterial chromosome. Science, 342(6159), 731–734. Available from: 10.1126/science.1242059 24158908 PMC3927313

[mmi15250-bib-0096] Li, S. , Liu, Q. , Duan, C. , Li, J. , Sun, H. , Xu, L. et al. (2023) c‐di‐GMP inhibits the DNA binding activity of H‐NS in *Salmonella* . Nature Communications, 14(1), 7502. Available from: 10.1038/s41467-023-43442-5 PMC10657408 37980414

[mmi15250-bib-0097] Lieberman‐Aiden, E. , van Berkum, N.L. , Williams, L. , Imakaev, M. , Ragoczy, T. , Telling, A. et al. (2009) Comprehensive mapping of long‐range interactions reveals folding principles of the human genome. Science, 326(5950), 289–293. Available from: 10.1126/science.1181369 19815776 PMC2858594

[mmi15250-bib-0098] Lioy, V.S. , Cournac, A. , Marbouty, M. , Duigou, S. , Mozziconacci, J. , Espéli, O. et al. (2018) Multiscale structuring of the *E. coli* chromosome by nucleoid‐associated and condensin proteins. Cell, 172(4), 771–783.e18. Available from: 10.1016/j.cell.2017.12.027 29358050

[mmi15250-bib-0099] Liu, L. , Wang, G. , Song, L. , Lv, B. & Liang, W. (2016) Acetylome analysis reveals the involvement of lysine acetylation in biosynthesis of antibiotics in *Bacillus amyloliquefaciens* . Scientific Reports, 6(1), 20108. Available from: 10.1038/srep20108 26822828 PMC4731788

[mmi15250-bib-0100] Liu, Y. , Chen, H. , Kenney, L.J. & Yan, J. (2010) A divalent switch drives H‐NS/DNA‐binding conformations between stiffening and bridging modes. Genes & Development, 24(4), 339–344. Available from: 10.1101/gad.1883510 20159954 PMC2816733

[mmi15250-bib-0101] Lourenço, R.F. , Saurabh, S. , Herrmann, J. , Wakatsuki, S. & Shapiro, L. (2020) The nucleoid‐associated protein GapR uses conserved structural elements to oligomerize and bind DNA. MBio, 11(3), e00448‐20. Available from: 10.1128/mBio.00448-20 32518183 PMC7373187

[mmi15250-bib-0102] Luijsterburg, M.S. , Noom, M.C. , Wuite, G.J.L. & Dame, R.T. (2006) The architectural role of nucleoid‐associated proteins in the organization of bacterial chromatin: a molecular perspective. Journal of Structural Biology, 156(2), 262–272. Available from: 10.1016/j.jsb.2006.05.006 16879983

[mmi15250-bib-0103] Luijsterburg, M.S. , White, M.F. , van Driel, R. & Dame, R.T. (2008) The major architects of chromatin: architectural proteins in bacteria, archaea and eukaryotes. Critical Reviews in Biochemistry and Molecular Biology, 43(6), 393–418. Available from: 10.1080/10409230802528488 19037758

[mmi15250-bib-0104] Macek, B. , Mijakovic, I. , Olsen, J.V. , Gnad, F. , Kumar, C. , Jensen, P.R. et al. (2007) The serine/threonine/tyrosine phosphoproteome of the model bacterium *Bacillus subtilis* . Molecular & Cellular Proteomics, 6(4), 697–707. Available from: 10.1074/mcp.M600464-MCP200 17218307

[mmi15250-bib-0105] Marbouty, M. , Le Gall, A. , Cattoni, D.I. , Cournac, A. , Koh, A. , Fiche, J.‐B. et al. (2015) Condensin‐ and replication‐mediated bacterial chromosome folding and origin condensation revealed by hi‐C and super‐resolution imaging. Molecular Cell, 59(4), 588–602. Available from: 10.1016/J.MOLCEL.2015.07.020 26295962

[mmi15250-bib-0106] Marciniak, B.C. , Trip, H. , Fusetti, F. & Kuipers, O.P. (2012) Regulation of *ykrL* (*htpX*) by Rok and YkrK, a novel type of regulator in *Bacillus subtilis* . Journal of Bacteriology, 194(11), 2837–2845. Available from: 10.1128/JB.00324-12 22447908 PMC3370632

[mmi15250-bib-0107] Melby, T.E. , Ciampaglio, C.N. , Briscoe, G. & Erickson, H.P. (1998) The symmetrical structure of structural maintenance of chromosomes (SMC) and MukB proteins: long, antiparallel coiled coils, folded at a flexible hinge. The Journal of Cell Biology, 142(6), 1595–1604. Available from: 10.1083/JCB.142.6.1595 9744887 PMC2141774

[mmi15250-bib-0108] Micka, B. , Groch, N. , Heinemann, U. & Marahiel, M.A. (1991) Molecular cloning, nucleotide sequence, and characterization of the *Bacillus subtilis* gene encoding the DNA‐binding protein HBsu. Journal of Bacteriology, 173(10), 3191–3198. Available from: 10.1128/jb.173.10.3191-3198.1991 1902464 PMC207914

[mmi15250-bib-0109] Micka, B. & Marahiel, M. (1992) The DNA‐binding protein HBsu is essential for normal growth and development in *Bacillus subtilis* . Biochimie, 74(7–8), 641–650. Available from: 10.1016/0300-9084(92)90136-3 1382620

[mmi15250-bib-0110] Mirabella, A. , Terwagne, M. , Zygmunt, M.S. , Cloeckaert, A. , De Bolle, X. & Letesson, J.J. (2013) *Brucella melitensis* MucR, an orthologue of *Sinorhizobium meliloti* MucR, is involved in resistance to oxidative, detergent, and saline stresses and cell envelope modifications. Journal of Bacteriology, 195(3), 453–465. Available from: 10.1128/JB.01336-12 23161025 PMC3554010

[mmi15250-bib-0111] Moriya, S. , Tsujikawa, E. , Hassan, A.K.M. , Asai, K. , Kodama, T. & Ogasawara, N. (1998) A *Bacillus subtilis* gene‐encoding protein homologous to eukaryotic SMC motor protein is necessary for chromosome partition. Molecular Microbiology, 29(1), 179–187. Available from: 10.1046/j.1365-2958.1998.00919.x 9701812

[mmi15250-bib-0112] Nagarajavel, V. , Madhusudan, S. , Dole, S. , Rahmouni, A.R. & Schnetz, K. (2007) Repression by binding of H‐NS within the transcription unit. Journal of Biological Chemistry, 282(32), 23622–23630. Available from: 10.1074/jbc.M702753200 17569663

[mmi15250-bib-0113] Nicolas, P. (2002) Mining *Bacillus subtilis* chromosome heterogeneities using hidden Markov models. Nucleic Acids Research, 30(6), 1418–1426. Available from: 10.1093/nar/30.6.1418 11884641 PMC101363

[mmi15250-bib-0114] Niki, H. , Jaffe, A. , Imamura, R. , Ogura, T. & Hiraga, S. (1991) The new gene mukB codes for a 177 kd protein with coiled‐coil domains involved in chromosome partitioning of *E. coli* . The EMBO Journal, 10(1), 183–193. Available from: 10.1002/J.1460-2075.1991.TB07935.X 1989883 PMC452628

[mmi15250-bib-0115] Nora, E.P. , Lajoie, B.R. , Schulz, E.G. , Giorgetti, L. , Okamoto, I. , Servant, N. et al. (2012) Spatial partitioning of the regulatory landscape of the X‐inactivation centre. Nature, 485(7398), 381–385. Available from: 10.1038/nature11049 22495304 PMC3555144

[mmi15250-bib-0116] Odijk, T. (1998) Osmotic compaction of supercoiled DNA into a bacterial nucleoid. Biophysical Chemistry, 73(1–2), 23–29. Available from: 10.1016/S0301-4622(98)00115-X 9697298

[mmi15250-bib-0117] Ogura, Y. , Imai, Y. , Ogasawara, N. & Moriya, S. (2001) Autoregulation of the *dnaA‐dnaN* operon and effects of DnaA protein levels on replication initiation in *Bacillus subtilis* . Journal of Bacteriology, 183(13), 3833–3841. Available from: 10.1128/JB.183.13.3833-3841.2001 11395445 PMC95264

[mmi15250-bib-0118] Ono, S. , Goldberg, M.D. , Olsson, T. , Esposito, D. , Hinton, J.C.D. & Ladbury, J.E. (2005) H‐NS is a part of a thermally controlled mechanism for bacterial gene regulation. The Biochemical Journal, 391(Pt 2), 203–213. Available from: 10.1042/BJ20050453 15966862 PMC1276917

[mmi15250-bib-0119] Palmieri, M. , Malgieri, G. , Russo, L. , Baglivo, I. , Esposito, S. , Netti, F. et al. (2013) Structural Zn(II) implies a switch from fully cooperative to partly downhill folding in highly homologous proteins. Journal of the American Chemical Society, 135(13), 5220–5228. Available from: 10.1021/ja4009562 23484956

[mmi15250-bib-0120] Pan, C.Q. , Finkel, S.E. , Cramton, S.E. , Feng, J.A. , Sigman, D.S. & Johnson, R.C. (1996) Variable structures of Fis‐DNA complexes determined by flanking DNA‐protein contacts. Journal of Molecular Biology, 264(4), 675–695. Available from: 10.1006/jmbi.1996.0669 8980678

[mmi15250-bib-0121] Pirone, L. , Pitzer, J.E. , D'Abrosca, G. , Fattorusso, R. , Malgieri, G. , Pedone, E.M. et al. (2018) Identifying the region responsible for *Brucella abortus* MucR higher‐order oligomer formation and examining its role in gene regulation. Scientific Reports, 8(1), 17238. Available from: 10.1038/s41598-018-35432-1 30467359 PMC6250670

[mmi15250-bib-0122] Qin, L. , Bdira, F.B. , Sterckx, Y.G.J. , Volkov, A.N. , Vreede, J. , Giachin, G. et al. (2020) Structural basis for osmotic regulation of the DNA binding properties of H‐NS proteins. Nucleic Acids Research, 48(4), 2156–2172. Available from: 10.1093/nar/gkz1226 31925429 PMC7039000

[mmi15250-bib-0123] Qin, L. , Erkelens, A.M. , Ben Bdira, F. & Dame, R.T. (2019) The architects of bacterial DNA bridges: a structurally and functionally conserved family of proteins. Open Biology, 9(12), 190223. Available from: 10.1098/rsob.190223 31795918 PMC6936261

[mmi15250-bib-0124] Qu, Y. , Lim, C.J. , Whang, Y.R. , Liu, J. & Yan, J. (2013) Mechanism of DNA organization by *Mycobacterium tuberculosis* protein Lsr2. Nucleic Acids Research, 41(10), 5263–5272. Available from: 10.1093/nar/gkt249 23580555 PMC3664827

[mmi15250-bib-0125] Racki, L.R. , Tocheva, E.I. , Dieterle, M.G. , Sullivan, M.C. , Jensen, G.J. & Newman, D.K. (2017) Polyphosphate granule biogenesis is temporally and functionally tied to cell cycle exit during starvation in *Pseudomonas aeruginosa* . Proceedings of the National Academy of Sciences, 114(12), E2440–E2449. Available from: 10.1073/pnas.1615575114 PMC5373386 28265086

[mmi15250-bib-0126] Rao, S.S.P. , Huntley, M.H. , Durand, N.C. , Stamenova, E.K. , Bochkov, I.D. , Robinson, J.T. et al. (2014) A 3D map of the human genome at kilobase resolution reveals principles of chromatin looping. Cell, 159(7), 1665–1680. Available from: 10.1016/J.CELL.2014.11.021 25497547 PMC5635824

[mmi15250-bib-0127] Rashid, F.‐Z.M. , Crémazy, F.G.E. , Hofmann, A. , Forrest, D. , Grainger, D.C. , Heermann, D.W. et al. (2023) The environmentally‐regulated interplay between local three‐dimensional chromatin organisation and transcription of *proVWX* in *E. Coli*. *Nature* . Communications, 14(1), 7478. Available from: 10.1038/s41467-023-43322-y PMC10656529 37978176

[mmi15250-bib-0128] Reverdy, A. , Chen, Y. , Hunter, E. , Gozzi, K. & Chai, Y. (2018) Protein lysine acetylation plays a regulatory role in *Bacillus subtilis* multicellularity. PLoS One, 13(9), e0204687. Available from: 10.1371/journal.pone.0204687 30265683 PMC6161898

[mmi15250-bib-0129] Ricci, D.P. , Melfi, M.D. , Lasker, K. , Dill, D.L. , McAdams, H.H. & Shapiro, L. (2016) Cell cycle progression in *Caulobacter* requires a nucleoid‐associated protein with high AT sequence recognition. Proceedings of the National Academy of Sciences of the United States of America, 113(40), E5952–E5961. Available from: 10.1073/pnas.1612579113 27647925 PMC5056096

[mmi15250-bib-0130] Rice, P.A. , Yang, S. , Mizuuchi, K. & Nash, H.A. (1996) Crystal structure of an IHF‐DNA complex: a protein‐induced DNA U‐turn. Cell, 87(7), 1295–1306. Available from: 10.1016/S0092-8674(00)81824-3 8980235

[mmi15250-bib-0131] Roberts, D.M. (2023) A new role for monomeric ParA/Soj in chromosome dynamics in *Bacillus subtilis* . Microbiology, 12(1), e1344. Available from: 10.1002/mbo3.1344 PMC9841721 36825885

[mmi15250-bib-0132] Roberts, D.M. , Anchimiuk, A. , Kloosterman, T.G. , Murray, H. , Wu, L.J. , Gruber, S. et al. (2022) Chromosome remodelling by SMC/condensin in *B. subtilis* is regulated by monomeric Soj/ParA during growth and sporulation. Proceedings of the National Academy of Sciences of the United States of America, 119(41), e2204042119. Available from: 10.1073/pnas.2204042119 36206370 PMC9564211

[mmi15250-bib-0133] Rodriguez Ayala, F. , Bartolini, M. & Grau, R. (2020) The stress‐responsive alternative sigma factor SigB of *Bacillus subtilis* and its relatives: an old friend with new functions. Frontiers in Microbiology, 11, 555527. Available from: 10.3389/fmicb.2020.01761 PMC7522486 33042030

[mmi15250-bib-0134] Russo, L. , Palmieri, M. , Baglivo, I. , Esposito, S. , Isernia, C. , Malgieri, G. et al. (2010) NMR assignments of the DNA binding domain of Ml4 protein from *Mesorhizobium loti* . Biomolecular NMR Assignments, 4(1), 55–57. Available from: 10.1007/s12104-009-9206-0 20020226

[mmi15250-bib-0135] Sanborn, A.L. , Rao, S.S.P. , Huang, S.‐C. , Durand, N.C. , Huntley, M.H. , Jewett, A.I. et al. (2015) Chromatin extrusion explains key features of loop and domain formation in wild‐type and engineered genomes. Proceedings of the National Academy of Sciences of the United States of America, 112(47), E6456–E6465. Available from: 10.1073/pnas.1518552112 26499245 PMC4664323

[mmi15250-bib-0136] Sanulli, S. , Trnka, M.J. , Dharmarajan, V. , Tibble, R.W. , Pascal, B.D. , Burlingame, A.L. et al. (2019) HP1 reshapes nucleosome core to promote phase separation of heterochromatin. Nature, 575(7782), 390–394. Available from: 10.1038/S41586-019-1669-2 31618757 PMC7039410

[mmi15250-bib-0137] Schultz, D. , Wolynes, P.G. , Jacob, E.B. & Onuchic, J.N. (2009) Deciding fate in adverse times: sporulation and competence in *Bacillus subtilis* . Proceedings of the National Academy of Sciences of the United States of America, 106(50), 21027–21034. Available from: 10.1073/pnas.0912185106 19995980 PMC2795487

[mmi15250-bib-0138] Seid, C.A. , Smith, J.L. & Grossman, A.D. (2017) Genetic and biochemical interactions between the bacterial replication initiator DnaA and the nucleoid‐associated protein Rok in *Bacillus subtilis* . Molecular Microbiology, 103(5), 798–817. Available from: 10.1111/mmi.13590 27902860 PMC5323315

[mmi15250-bib-0139] Sekimizu, K. , Bramhill, D. & Kornberg, A. (1987) ATP activates dnaA protein in initiating replication of plasmids bearing the origin of the *E. coli* chromosome. Cell, 50(2), 259–265. Available from: 10.1016/0092-8674(87)90221-2 3036372

[mmi15250-bib-0140] Serrano, E. , Torres, R. & Alonso, J.C. (2021) Nucleoid‐associated Rok differentially affects chromosomal transformation on *Bacillus subtilis* recombination‐deficient cells. Environmental Microbiology, 23(6), 3318–3331. Available from: 10.1111/1462-2920.15562 33973337

[mmi15250-bib-0141] Serror, P. & Sonenshein, A.L. (1996) CodY is required for nutritional repression of *Bacillus subtilis* genetic competence. Journal of Bacteriology, 178(20), 5910–5915. Available from: 10.1128/jb.178.20.5910-5915.1996 8830686 PMC178446

[mmi15250-bib-0142] Sexton, T. , Yaffe, E. , Kenigsberg, E. , Bantignies, F. , Leblanc, B. , Hoichman, M. et al. (2012) Three‐dimensional folding and functional organization principles of the *Drosophila* genome. Cell, 148(3), 458–472. Available from: 10.1016/j.cell.2012.01.010 22265598

[mmi15250-bib-0143] Shi, W.‐T. , Zhang, B. , Li, M.‐L. , Liu, K.‐H. , Jiao, J. & Tian, C.‐F. (2022) The convergent xenogeneic silencer MucR predisposes α‐proteobacteria to integrate AT‐rich symbiosis genes. Nucleic Acids Research, 50(15), 8580–8598. Available from: 10.1093/nar/gkac664 36007892 PMC9410896

[mmi15250-bib-0144] Shin, Y. & Brangwynne, C.P. (2017) Liquid phase condensation in cell physiology and disease. Science, 357(6357), eaaf4382. Available from: 10.1126/science.aaf4382 28935776

[mmi15250-bib-0145] Singh, K. , Milstein, J.N. & Navarre, W.W. (2016) Xenogeneic silencing and its impact on bacterial genomes. Annual Review of Microbiology, 70(1), 199–213. Available from: 10.1146/annurev-micro-102215-095301 27359215

[mmi15250-bib-0146] Singh, P.K. , Ramachandran, G. , Durán‐Alcalde, L. , Alonso, C. , Wu, L.J. & Meijer, W.J.J. (2012) Inhibition of *Bacillus subtilis* natural competence by a native, conjugative plasmid‐encoded *comK* repressor protein. Environmental Microbiology, 14(10), 2812–2825. Available from: 10.1111/j.1462-2920.2012.02819.x 22779408

[mmi15250-bib-0147] Smith, J.L. & Grossman, A.D. (2015) *In vitro* whole genome DNA binding analysis of the bacterial replication initiator and transcription factor DnaA. PLoS Genetics, 11(5), e1005258. Available from: 10.1371/journal.pgen.1005258 26020636 PMC4447404

[mmi15250-bib-0148] Smits, W.K. & Grossman, A.D. (2010) The transcriptional regulator Rok binds a+T‐rich DNA and is involved in repression of a Mobile genetic element in *Bacillus subtilis* . PLoS Genetics, 6(11), e1001207. Available from: 10.1371/journal.pgen.1001207 21085634 PMC2978689

[mmi15250-bib-0149] Smits, W.K. , Hoa, T.T. , Hamoen, L.W. , Kuipers, O.P. & Dubnau, D. (2007) Antirepression as a second mechanism of transcriptional activation by a minor groove binding protein. Molecular Microbiology, 64(2), 368–381. Available from: 10.1111/j.1365-2958.2007.05662.x 17493123 PMC3831528

[mmi15250-bib-0150] Soppa, J. , Kobayashi, K. , Noirot‐Gros, M. , Oesterhelt, D. , Ehrlich, S.D. , Dervyn, E. et al. (2002) Discovery of two novel families of proteins that are proposed to interact with prokaryotic SMC proteins, and characterization of the *Bacillus subtilis* family members ScpA and ScpB. Molecular Microbiology, 45(1), 59–71. Available from: 10.1046/j.1365-2958.2002.03012.x 12100548

[mmi15250-bib-0151] Stuger, R. , Woldringh, C.L. , van der Weijden, C.C. , Vischer, N.O. , Bakker, B.M. , van Spanning, R.J. et al. (2002) DNA supercoiling by gyrase is linked to nucleoid compaction. Molecular Biology Reports, 29(1–2), 79–82. Available from: 10.1023/a:1020318705894 12241080

[mmi15250-bib-0152] Sullivan, N.L. , Marquis, K.A. & Rudner, D.Z. (2009) Recruitment of SMC by ParB‐parS organizes the origin region and promotes efficient chromosome segregation. Cell, 137(4), 697–707. Available from: 10.1016/J.CELL.2009.04.044 19450517 PMC2892783

[mmi15250-bib-0153] Summers, E.L. , Meindl, K. , Usón, I. , Mitra, A.K. , Radjainia, M. , Colangeli, R. et al. (2012) The structure of the Oligomerization domain of Lsr2 from *Mycobacterium tuberculosis* reveals a mechanism for chromosome organization and protection. PLoS One, 7(6), e38542. Available from: 10.1371/journal.pone.0038542 22719899 PMC3374832

[mmi15250-bib-0154] Suzuki‐Minakuchi, C. , Kawazuma, K. , Matsuzawa, J. , Vasileva, D. , Fujimoto, Z. , Terada, T. et al. (2016) Structural similarities and differences in H‐NS family proteins revealed by the N‐terminal structure of TurB in *Pseudomonas putida* KT2440. FEBS Letters, 590(20), 3583–3594. Available from: 10.1002/1873-3468.12425 27709616

[mmi15250-bib-0155] Swinger, K.K. , Lemberg, K.M. , Zhang, Y. & Rice, P.A. (2003) Flexible DNA bending in HU‐DNA cocrystal structures. EMBO Journal, 22(14), 3749–3760. Available from: 10.1093/emboj/cdg351 12853489 PMC165621

[mmi15250-bib-0156] Szabo, Q. , Bantignies, F. & Cavalli, G. (2019) Principles of genome folding into topologically associating domains. Science Advances, 5(4), eaaw1668. Available from: 10.1126/SCIADV.AAW1668 30989119 PMC6457944

[mmi15250-bib-0157] Tarry, M.J. , Harmel, C. , Taylor, J.A. , Marczynski, G.T. & Schmeing, T.M. (2019) Structures of GapR reveal a central channel which could accommodate B‐DNA. Scientific Reports, 9(1), 16679. Available from: 10.1038/S41598-019-52964-2 31723182 PMC6853979

[mmi15250-bib-0158] Tendeng, C. , Soutourina, O.A. , Danchin, A. & Bertin, P.N. (2003) MvaT proteins in *Pseudomonas* spp.: a novel class of H‐NS‐like proteins. Microbiology, 149(11), 3047–3050. Available from: 10.1099/mic.0.C0125-0 14600217

[mmi15250-bib-0159] Tišma, M. , Bock, F.P. , Kerssemakers, J. , Japaridze, A. , Gruber, S. & Dekker, C. (2023) Direct observation of a crescent‐shape chromosome in *Bacillus subtilis* . BioRxiv 10.1101/2023.02.09.527813 PMC10979009 38548820

[mmi15250-bib-0160] Ueguchi, C. & Mizuno, T. (1993) The *Escherichia coli* nucleoid protein H‐NS functions directly as a transcriptional repressor. The EMBO Journal, 12(3), 1039–1046. Available from: 10.1002/j.1460-2075.1993.tb05745.x 8458322 PMC413305

[mmi15250-bib-0161] van Berkum, N.L. , Lieberman‐Aiden, E. , Williams, L. , Imakaev, M. , Gnirke, A. , Mirny, L.A. et al. (2010) Hi‐C: a method to study the three‐dimensional architecture of genomes. Journal of Visualized Experiments, 39, 1869. Available from: 10.3791/1869 PMC3149993 20461051

[mmi15250-bib-0162] van der Valk, R.A. , Vreede, J. , Qin, L. , Moolenaar, G.F. , Hofmann, A. , Goosen, N. et al. (2017) Mechanism of environmentally driven conformational changes that modulate H‐NS DNA‐bridging activity. eLife, 6, e27369. Available from: 10.7554/eLife.27369 28949292 PMC5647153

[mmi15250-bib-0163] Wang, L. , Gao, Y. , Zheng, X. , Liu, C. , Dong, S. , Li, R. et al. (2019) Histone modifications regulate chromatin compartmentalization by contributing to a phase separation mechanism. Molecular Cell, 76(4), 646–659.e6. Available from: 10.1016/J.MOLCEL.2019.08.019 31543422

[mmi15250-bib-0164] Wang, Q. , Sun, Q. , Czajkowsky, D.M. & Shao, Z. (2018) Sub‐kb Hi‐C in *D. melanogaster* reveals conserved characteristics of TADs between insect and mammalian cells. Nature Communications, 9(1), 188. Available from: 10.1038/s41467-017-02526-9 PMC5768742 29335463

[mmi15250-bib-0165] Wang, S. , Dong, X. , Zhu, Y. , Wang, C. , Sun, G. , Luo, T. et al. (2013) Revealing of *Mycobacterium marinum* transcriptome by RNA‐seq. PLoS One, 8(9), e75828. Available from: 10.1371/journal.pone.0075828 24098731 PMC3786904

[mmi15250-bib-0166] Wang, X. , Tang, O.W. , Riley, E.P. & Rudner, D.Z. (2014) The SMC condensin complex is required for origin segregation in *Bacillus subtilis* . Current Biology, 24(3), 287–292. Available from: 10.1016/j.cub.2013.11.050 24440393 PMC3947903

[mmi15250-bib-0167] White, S.W. , Appelt, K. , Wilson, K.S. & Tanaka, I. (1989) A protein structural motif that bends DNA. Proteins, 5(4), 281–288. Available from: 10.1002/PROT.340050405 2508086

[mmi15250-bib-0168] White‐Ziegler, C.A. & Davis, T.R. (2009) Genome‐wide identification of H‐NS‐controlled, temperature‐regulated genes in *Escherichia coli* K‐12. Journal of Bacteriology, 191(3), 1106–1110. Available from: 10.1128/JB.00599-08 19011022 PMC2632076

[mmi15250-bib-0169] Wiechert, J. , Filipchyk, A. , Hünnefeld, M. , Gätgens, C. , Brehm, J. , Heermann, R. et al. (2020) Deciphering the rules underlying xenogeneic silencing and counter‐silencing of Lsr2‐like proteins using CgpS of *Corynebacterium glutamicum* as a model. MBio, 11(1), e02273‐19. Available from: 10.1128/mBio.02273-19 32019787 PMC7002338

[mmi15250-bib-0170] Winardhi, R.S. , Fu, W. , Castang, S. , Li, Y. , Dove, S.L. & Yan, J. (2012) Higher order oligomerization is required for H‐NS family member MvaT to form gene‐silencing nucleoprotein filament. Nucleic Acids Research, 40(18), 8942–8952. Available from: 10.1093/nar/gks669 22798496 PMC3467065

[mmi15250-bib-0171] Winardhi, R.S. , Yan, J. & Kenney, L.J. (2015) H‐NS regulates gene expression and compacts the nucleoid: insights from single‐molecule experiments. Biophysical Journal, 109(7), 1321–1329. Available from: 10.1016/j.bpj.2015.08.016 26445432 PMC4601063

[mmi15250-bib-0172] Yadav, T. , Carrasco, B. , Myers, A.R. , George, N.P. , Keck, J.L. & Alonso, J.C. (2012) Genetic recombination in *Bacillus subtilis*: a division of labor between two single‐strand DNA‐binding proteins. Nucleic Acids Research, 40(12), 5546–5559. Available from: 10.1093/nar/gks173 22373918 PMC3384303

